# Exportin 1 modulates life span by regulating nucleolar dynamics via the autophagy protein LGG-1/GABARAP

**DOI:** 10.1126/sciadv.abj1604

**Published:** 2022-04-01

**Authors:** Anita V. Kumar, Taewook Kang, Tara G. Thakurta, Celeste Ng, Aric N. Rogers, Martin R. Larsen, Louis R. Lapierre

**Affiliations:** 1 Department of Molecular Biology, Cell Biology, and Biochemistry, Brown University, 185 Meeting St., Providence, RI 02912, USA.; 2 Department of Biochemistry and Molecular Biology, University of Southern Denmark, Odense, Denmark.; 3 MDI Biological Laboratory, 159 Old Bar Harbor Rd., Salisbury Cove, ME 04672, USA.

## Abstract

Altered nucleolar and ribosomal dynamics are key hallmarks of aging, but their regulation remains unclear. Building on the knowledge that the conserved nuclear export receptor Exportin 1 (XPO-1/XPO1) modulates proteostasis and life span, we systematically analyzed the impact of nuclear export on protein metabolism. Using transcriptomic and subcellular proteomic analyses in nematodes, we demonstrate that XPO-1 modulates the nucleocytoplasmic distribution of key proteins involved in nucleolar dynamics and ribosome function, including fibrillarin (FIB-1/FBL) and RPL-11 (RPL11). Silencing *xpo-1* led to marked reduction in global translation, which was accompanied by decreased nucleolar size and lower fibrillarin levels. A targeted screen of known proteostatic mediators revealed that the autophagy protein LGG-1/GABARAP modulates nucleolar size by regulating RPL-11 levels, linking specific protein degradation to ribosome metabolism. Together, our study reveals that nucleolar size and life span are regulated by LGG-1/GABARAP via ribosome protein surveillance.

## Teaser

Proteostasis and nucleolar dynamics are coordinated by Exportin 1 to modulate life span.

## INTRODUCTION

In eukaryotic cells, compartmentalization separates genomic DNA in the nucleus from the rest of the cell by the nuclear envelope. Export of several proteins and RNA from the nucleus to the cytoplasm depends on highly conserved export machinery consisting of the nuclear pore complex (NPC), export receptors, and metabolic energy exchange complexes (*1*). A highly conserved nuclear export receptor, Exportin 1 (XPO1/CRM1), binds cargo proteins containing nuclear export signal sequences and facilitates their transport through the NPC in a Ran–guanosine triphosphate–dependent manner (*2*). Functional and proteomics studies have revealed that XPO1 exports a wide array of over 200 proteins including 40*S* and 60*S* preribosomal subunits, translation factors, vesicle coat proteins, cytoskeletal proteins, protein kinases, and transcription factors, many of which are tumor suppressors and oncoproteins (*3*, *4*). Therefore, alterations in XPO1 levels and activity could have ramifications on a wide array of processes ranging from ribosome biogenesis to oncogenesis and proteostasis.

Ribosome biogenesis is a multistep, energetically demanding process that initiates in the nucleolus with ribosomal RNA (rRNA) synthesis and processing and culminates in the cytoplasm with ribosome assembly and maturation. Ribosome biogenesis and protein synthesis affect overall cellular health and proteostasis by controlling cotranslational protein folding, translational fidelity, and the type of mRNAs being translated (*5*). Modest suppression of protein synthesis achieved by mutations in ribosomal subunits and translation factors increases life span in yeast, nematodes, and fruitflies (*6*–*8*). This longevity effect has been attributed to reduced burden on the proteostasis machinery (*9*), reduced protein synthesis rates that lead to higher translation accuracy (*10*, *11*), and, possibly, reallocation of cellular energy toward processes such as protein folding and degradation (*12*).

Recently, our laboratory demonstrated that inhibition of XPO-1/XPO1 extends the mean life span of *Caenorhabditis elegans* by about 15 to 45% (*13*). Reducing the activity of XPO-1/XPO1 in nematodes and human HeLa cells leads to nuclear accumulation of TFEB (transcription factor EB; known as HLH-30 in nematodes), a master regulator of autophagy and lysosome biogenesis (*13*, *14*). We observed increased autophagy, lysosomes, protein aggregate clearance, and enhanced life span upon genetic and pharmacological XPO1 inhibition (*13*). Since XPO1 is known to facilitate nuclear export of a large protein repertoire including transcription factors and preribosomal subunits (*15*), in addition to TFEB, we predicted that XPO1 modulation would potentially influence transport of several proteins across the nucleus, leading to global alterations in proteostasis. To test this, we used a multi-omics approach studying the transcriptome, translatome, and nuclear and non-nuclear proteomes to identify cellular physiological alterations brought about by XPO-1 inhibition. We identified nucleolar protein, fibrillarin (FIB-1/FBL), and large ribosomal subunit protein, RPL-11.1/RPL11, as key players in XPO-1–mediated modulation of nucleolar size and longevity. Specifically, the HLH-30/TFEB–regulated protein, LGG-1/GABARAP (GABA type A receptor-associated protein), modulates nucleolar size by directly controlling RPL-11 levels, which substantially improved proteostasis and life span. Together, our work demonstrates cross-talk between nucleolar dynamics, proteostasis, and the rate of nuclear export, thus uncovering a previously unidentified regulatory mechanism between convergent hallmarks of longevity.

## RESULTS

### *xpo-1* silencing results in transcriptional down-regulation and nuclear repartitioning of ribosomal genes and translation initiation factors

**Fig. 1.**
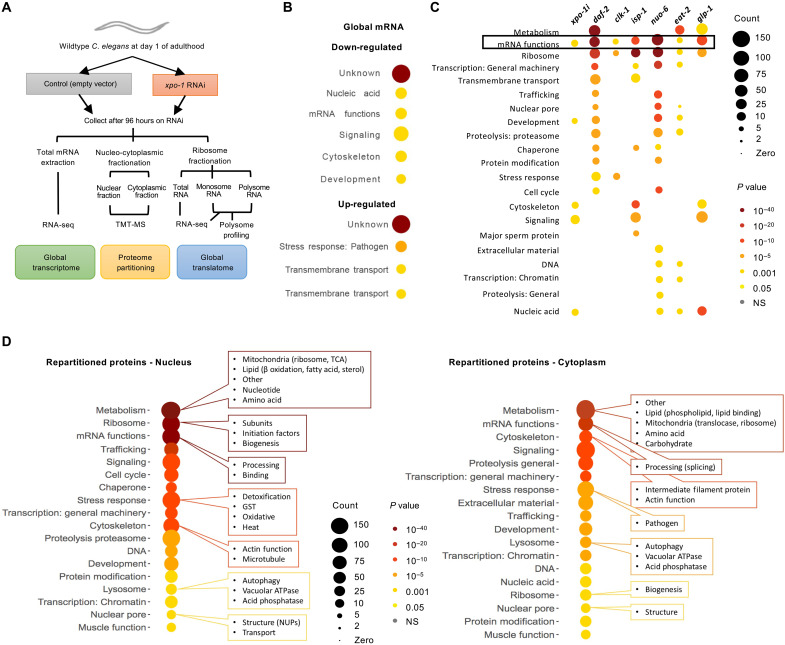
*xpo-1* silencing down-regulates expression of genes involved in protein synthesis and alters nucleocytoplasmic protein partitioning. (**A**) Schematic illustrating the omics approaches taken to study molecular changes in nematodes with nuclear export inhibition. (**B**) GO categories of differentially regulated genes significantly enriched upon *xpo-1* RNAi compared to control after 96 hours using WormCat database (*16*). See data file S1 for complete list of significant differentially regulated transcripts and fig. S1 (A to C) for principal components analysis (PCA), volcano plots, and heatmaps. (**C**) Comparison of GO categories of significantly down-regulated genes from long-lived mutants (*17*, *18*) with those for *xpo-1* RNAi from this study. (**D**) Protein GO categories and subcategories that are either enriched in nuclear and non-nuclear (cytoplasmic) fractions after 96 hours of *xpo-1* RNAi compared to control. See fig. S1D for purity of fractions and data file S2 for complete list of significant repartitioned proteins. NS, not significant.

Nuclear export inhibition by silencing *xpo-1* enhanced autophagy, proteostatic benefits, and increased life span (*13*). To characterize molecular changes in nematodes with inhibited nuclear export, we performed total mRNA sequencing on wild-type nematodes with RNA interference (RNAi)–mediated silencing of *xpo-1* for 96 hours from adulthood (Fig. 1A). RNA sequencing (RNA-seq) revealed 2553 significantly differentiated transcripts of which 1172 were down-regulated and 1381 were up-regulated (data file S1 and fig. S1, A to C). Gene ontology (GO) analysis using WormCat (*16*) revealed that transcripts related to mRNA function including nucleolar proteins were significantly down-regulated while those related to stress response were up-regulated (Fig. 1B). Comparison of this down-regulated gene set with those from known long-lived mutant nematodes such as *daf-2(e1370)*, *clk-1(qm30)*, *isp-1(qm150)*, *nuo-6(qm200)*, *eat-2(ad465)*, and *glp-1(e2141)* from published datasets (*17*, *18*) revealed that mRNA functions were down-regulated across longevity mutants irrespective of the mutation conferring longevity (Fig. 1C). As XPO-1 is a major nuclear protein export receptor, we next examined nuclear and cytoplasmic (non-nuclear) proteomes from *xpo-1*–silenced nematodes compared to control (Fig. 1A and fig. S1D). Tandem mass tagging of peptides followed by mass spectrometry (TMT-MS) analyses revealed major changes in partitioning of proteins across the nucleus upon *xpo-1* RNAi (data file S2). When the average log_2_ intensity ratio for nuclear fraction over cytoplasmic fraction in *xpo-1* RNAi versus control was >1, the protein was considered nuclear repartitioned, while a ratio <1 was cytoplasm repartitioned. This method identifies relative changes in nucleocytoplasmic distribution irrespective of overall changes in protein levels but does not differentiate between changes in nucleocytoplasmic transport and protein turnover. Therefore, a change in partitioning could either stem from change in nuclear export or from altered protein turnover in the nucleus or cytoplasm. Proteins with nucleus over cytoplasm log_2_ intensity ratios that were statistically significant were considered for further analysis. GO term analysis using WormCat (*16*) indicated that metabolism, ribosome, mRNA function, chaperone, and proteasome were the top categories repartitioned (Fig. 1D). Different subcategories of proteins within the parent GO term were either repartitioned in the nucleus or cytoplasm. For instance, in the category of ribosomes, nuclear repartitioning included ribosomal subunits, biogenesis factors, and translation initiation factors, while cytoplasmic repatriating included only biogenesis proteins (Fig. 1D). Overall, 3609 proteins were identified as nuclear repartitioned including proteins involved in lipid and nucleotide metabolism, binding and processing of mRNA, ribosome formation and function, proteasome-mediated degradation, oxidative and heat stress response, autophagy, and actin and microtubule cytoskeleton. Among the repartitioned proteins in the cytoplasm, 2152 proteins were identified including phospholipid and carbohydrate metabolism, mRNA splicing, few ribosome biogenesis factors, intermediate filament proteins, pathogen stress response factors, and autophagy and lysosomal function (Fig. 1D and data file S2). About 6% of the proteins identified as repartitioned were reported as potential XPO1 cargoes by (data file S2) (*15*), indicating likely previously unidentified cargoes and/or secondary effects of export inhibition–mediated protein repartitioning. Analysis of the overlaps between our transcriptomic and proteomic data identified proteins whose transcripts were differentially regulated in addition to being repartitioned including pathways such as mRNA functions, metabolism, development, and proteolysis (fig. S1E). Overall, our data point toward a reduction in ribosome biogenesis and translation machinery in the cytoplasm of *xpo-1*–silenced nematodes compared to control.

### *xpo-1* silencing reduces global translation but enhances translation of a subset of mRNA

**Fig. 2.**
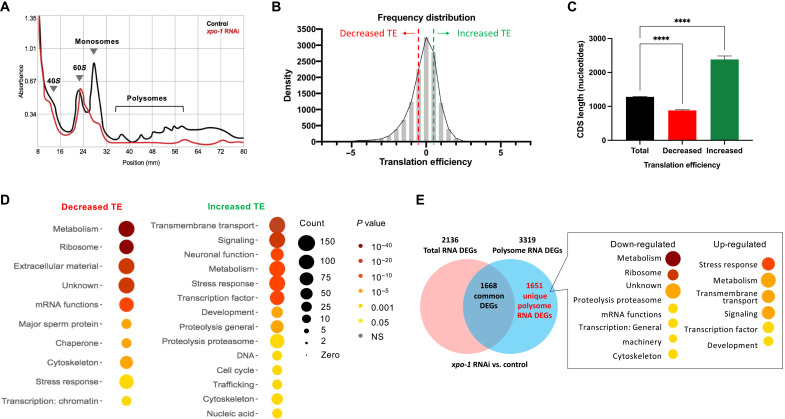
*xpo-1* silencing reduces global translation but enhances translation of a subset of mRNA. (**A**) Representative polysome profiles of ribosomes extracted from control and *xpo-1* RNAi nematodes after 96 hours on RNAi. (**B**) Histogram of genes with increased or decreased TE, calculated as log_2_ ratio of polysomal versus monosomal differences between *xpo-1* RNAi and control; calculation method adapted from (*69*). See data file S3 for complete list of proteins with altered TE and fig. S2 for PCA and volcano plots. (**C**) CDS length of transcripts with decreased (*n* = 881) or increased (*n* = 692) TE compared to total transcripts (*n* = 12048) [mean ± SEM, analysis of variance (ANOVA), ****P < 0.0001]. (**D**) WormCat GO categories of proteins with altered TE from (B). (**E**) Venn diagram depicting common and unique polysome differentially expressed genes (DEGs) in *xpo-1* RNAi compared to control and the WormCat GO categories represented by these unique DEGs. See data file S4 for complete list of unique polysome DEGs.

Owing to the observed down-regulation and nuclear repartitioning of ribosomal components with *xpo-1* silencing and the central role played by XPO-1 in preribosomal subunit export (*15*), we measured translation levels by polysome profiling in control and *xpo-1*–silenced nematodes. Polysome profiles revealed a marked flattening of monosome and polysome peaks with *xpo-1* silencing without an obvious change in the 40*S* and 60*S* peaks (Fig. 2A), indicating a global reduction in translation likely due to the observed transcriptional reduction and repartitioning of components related to ribosome and mRNA function. Sequencing of mRNA obtained from monosome and polysome fractions uncovered mRNAs with altered translation efficiency (TE; fig. S2, A and B). Notably, despite the reduction in global translation, we uncovered a subset of mRNA with increased TE (Fig. 2B) calculated by comparing polysome and monosome mRNA counts in *xpo-1* RNAi versus control (data file S3). Genes that showed decreased TE had relatively shorter lengths of mRNA coding sequences (CDSs), while genes with increased TE had longer CDS (Fig. 2C), which suggests mRNA structure as a contributor of altered translation in *xpo-1* RNAi conditions. mRNAs with decreased TE mainly belong to metabolism, extracellular matrix, ribosome, and mRNA function pathways, while those with increased TE are mainly involved in cell survival pathways such as neuronal function, stress response, and signaling (Fig. 2D). Similar observations of increased translation of stress response and nuclear-encoded mitochondrial genes in the presence of global translation reduction have been made in other longevity paradigms achieved by attenuating translation initiation factor eIF4G (*19*) or dietary restriction (*20*). Using the GenAge database for genes implicated in altering life span (*21*), we found that several known antilongevity genes displayed reduced TE upon *xpo-1* silencing. Although the distribution of pro- and antilongevity genes appeared to be equal across changes in TE, there was a higher percentage of antilongevity genes (83.78%) than prolongevity genes in the −2 to −4-fold range (fig. S2C). To determine genes that are exclusively regulated at the translational level and unaffected transcriptionally, we compared differentially regulated polysome mRNA with that of total mRNA from *xpo-1* RNAi versus control extracted before ribosome fractionation (data file S4). In line with the TE patterns, we observed translational down-regulation of ribosome and mRNA function genes and up-regulation of stress response mRNAs (Fig. 2E), likely indicative of selective translational output mediated by the activity of XPO-1. Therefore, the reduction and repartitioning of ribosome biogenesis proteins in *xpo-1* knockdown nematodes is accompanied by global translation reduction and an altered translatome with a prolongevity signature.

### Life-span modulation by XPO-1 is dependent on nucleolar dynamics and the nucleolar methyltransferase fibrillarin

**Fig. 3.**
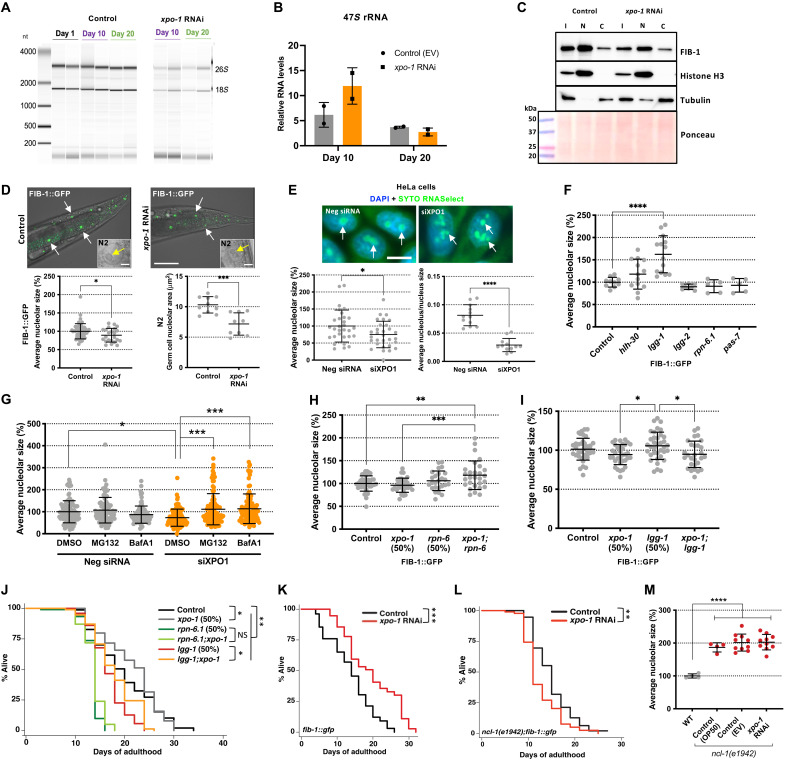
*xpo-1* life-span modulation links nucleolar dynamics to protein degradation systems. (**A**) rRNA levels (biological duplicates) on days 1, 10, and 20. (**B**) Expression of 47*S* pre-rRNA from control and *xpo-1* RNAi (biological duplicates). (**C**) FIB-1 protein levels in input (I), nuclear (N), and cytoplasmic (C) fractions after 96 hours of RNAi. Histone H3 and tubulin are markers for nuclear and cytoplasmic fractions, respectively. (**D**) Nucleoli (arrows) visualized by FIB-1::GFP and in germ cells in N2 (insets; scale bar, 5 μm) after 48 hours of RNAi (scale bar, 50 μm). Graphs represent average nucleolar size (% or μm^2^) from two to three independent experiments. (**E**) Nucleoli (arrows) in HeLa cells stained by SYTO RNASelect upon siRNA treatment for 48 hours. Graphs represent average nucleolar size (%) and nucleolar/nuclear size ratios from three independent experiments. (**F**) Average nucleolar size in FIB-1::GFP–expressing nematodes upon RNAi for autophagy and proteasomal genes for 48 hours. (**G**) Average nucleolar size in HeLa cells stained with SYTO RNASelect after treatment with siRNA for 48 hours along with MG132, bafilomycin A, or vehicle control dimethyl sulfoxide (DMSO). (**H** and **I**) Nucleolar size measurement with combined silencing of either *xpo-1* and *rpn-6.1* or *xpo-1* and *lgg-1* or 50% of individual RNAi in nematodes for 48 hours. (**J**) Life-span analyses in wild-type nematodes with *xpo-1*, *rpn-6.1*, and lgg-1 RNAi, alone or in combination with *xpo-1*. (**K** and **L**) Life-span analyses in *fib-1::gfp* (K) and *ncl-1(e1942);fib-1::gfp* (L) strains fed on control or *xpo-1* RNAi bacteria. (**M**) Average nucleolar size in *ncl-1(e1942);fib-1::gfp* strain on RNAi or OP50 for 48 hours compared to wild-type *fib-1::gfp* strain on OP50 (*n* = 4 to 10 nematodes). Data are represented as means ± SD from three independent trials. *P < 0.05, **P < 0.01, ***P < 0.001, and ****P < 0.0001 by Student’s *t* test or ANOVA or Mantel-Cox log rank test for life-span analyses. See table S1 for life-span statistics and repeats. DAPI, 4′,6-diamidino-2-phenylindole.

rRNA is transcribed, processed, and assembled into preribosomal subunits in the nucleolus (*22*), a nuclear compartment reported to be altered during aging (*23*). To determine how ribosome biogenesis may be modulated by XPO-1, we measured rRNA levels from *xpo-1*–silenced and control nematodes. We found a marked decrease in the intensity of 26*S* and 18*S* rRNA bands at days 10 and 20 of *xpo-1* RNAi (Fig. 3A). However, there was no significant change in the 47*S* pre-rRNA levels measured by quantitative polymerase chain reaction at the same time points (Fig. 3B), and this was confirmed using two additional primer sets for 47*S* rRNA from prior publications (fig. S3A) (*24*). Hence, we reasoned that the lower levels of mature rRNA found with *xpo-1* silencing could possibly stem from an attenuation of rRNA processing rather than a reduction in rRNA transcription. From our multi-omics datasets (data files S1 to S3), we identified the nucleolar rRNA methyltransferase, fibrillarin (FIB-1), to be transcriptionally and translationally down-regulated with higher repartitioning in the cytoplasm in *xpo-1* RNAi nematodes compared to control. FIB-1/FBL catalyzes 2′-*O*-methylation of ribose moieties in rRNA and is also involved in pre-rRNA processing (*25*). We confirmed the reduction of overall FIB-1/FBL levels upon *xpo-1/XPO1* silencing and observed that the cytoplasmic repartitioning was due to a decrease in nuclear FIB-1 levels (Fig. 3C and fig. S3B). Silencing *xpo-1* also resulted in smaller nucleolar size visualized by FIB-1::GFP (green fluorescent protein)–labeled nucleoli (*26*) and in wild-type (N2) nematodes and by SYTO RNASelect RNA staining in HeLa cells (Fig. 3, D and E), which corroborated the observed nuclear reduction of FIB-1 from our proteomics data. Nucleolar measurements in FIB-1::GFP nematodes with mutant *daf-2(e1370)* or *glp-1(e2141)* were used as positive controls and displayed smaller nucleoli as reported earlier (fig. S3C) (*23*). While these results demonstrate reduction in the size of FIB-1::GFP–tagged nucleoli with *xpo-1* RNAi, it must be acknowledged that overexpression of FIB-1 could have resulted in phenotypes that might not be found with endogenous FIB-1 levels. Therefore, we also used a CRISPR strain expressing nucleolar protein DAO-5 tagged to GFP (*27*). Similar to the results using FIB-1::GFP nematodes, we observed reduced nucleolar size in DAO-5::GFP–expressing nematodes (fig. S3D). RNA polymerase I inhibitor, CX-5461 (*28*), was used as a positive control for nucleolar disruption in HeLa cells (fig. S3E).

Reduced nucleolar size (*23*) and lower *xpo-1* levels (*13*) are common features of several long-lived nematode models, and most of these models display enhanced proteostatic capacity (*29*). Hence, we sought to investigate the link between proteostasis and nucleolar modulation in the context of *xpo-1* silencing. Knockdown of genes involved in autophagy and the ubiquitin-proteasome system showed alterations in nucleolar size with the most notable and significant change observed with silencing of *lgg-1* (Fig. 3F), which encodes an autophagosome membrane protein that is an ortholog of human GABARAP (*30*, *31*). Since *lgg-1* is a putative target gene for HLH-30 (*14*), we tested whether *lgg-1* transcription increased upon *xpo-1* RNAi in a HLH-30–dependent manner. As expected, loss of HLH-30 blunted the increase in *lgg-1* transcription upon *xpo-1* silencing; however, transcriptional changes in *fib-1* and 47*S* pre-rRNA were independent of HLH-30 loss (fig. S3F). In HeLa cells, silencing *GABARAP*, but not *LC3* (microtubule-associated proteins 1A/1B light chain 3B), also caused nucleolar expansion (fig. S3G). Moreover, we observed that blocking the proteasome with MG132 or blocking autophagy using the lysosome acidification inhibitor, bafilomycin A1, reversed the small-nucleoli phenotype brought about by *XPO1* silencing in HeLa cells (Fig. 3G), highlighting the significance of efficient proteostasis in modulating nucleolar size upon *xpo-1* depletion. Testing this in nematodes also showed a reversion of nucleolar size when the gene encoding longevity-associated proteasomal protein *rpn-6.1* (*32*) was silenced in addition to *xpo-1* (Fig. 3H). We also observed reversion of large nucleoli induced by *lgg-1* silencing when *xpo-1* and *lgg-1* were silenced together (Fig. 3I). Overall, these results suggest an active role for protein degradation pathways in nucleolar modulation.

Although low fibrillarin has been linked to longevity (*23*) and its partitioning is modulated by XPO-1 (Fig. 3C), the impact of the protein degradation system on nucleolar size and consequently, on life span, is unknown. To test this, we performed survival assays in wild-type and FIB-1–overexpressing nematodes and found that knockdown of *xpo-1* in addition to *rpn-6.1* or *lgg-1* silencing could not improve survival of *rpn-6.1* or *lgg-1* silencing conditions alone (Fig. 3J and fig. S3H). Moreover, *xpo-1* RNAi was unable to extend life span in the *rpn-10(ok1865)* strain (*33*) with a loss-of-function mutation in proteasomal protein RPN-10 (fig. S3I). Together, these indicated the requirement for functional proteasomes and autophagy machinery in XPO-1–mediated life-span modulation. Unexpectedly, we did not observe any accumulation of FIB-1 protein in nuclear or cytoplasmic fractions when *rpn-6.1* or *lgg-1* was silenced either alone or in addition to *xpo-1* silencing (fig. S4, A and B). This observation suggests that the reduction in FIB-1 levels when *xpo-1* is silenced mainly has a transcriptional origin, which we observed in our RNA-seq dataset (data file S1). *xpo-1* silencing extended life span in FIB-1–overexpressing nematodes in which nucleolar size is reduced by *xpo-1* RNAi (Fig. 3, K and D); however, this effect was completely lost in mutants of the FIB-1 translational repressor, NCL-1 (abnormal nucleoli-1) (*34*), in the FIB-1 overexpression background in which *xpo-1* RNAi is unable to reduce FIB-1 and nucleolar size because of the upstream *ncl-1* mutation (Fig. 3, L and M). This directly implicates nucleolar size reduction and FIB-1 modulation as mechanistic foundations for the effect of XPO-1 on life span. Together, these data highlight the requirement for enhanced proteostasis and nucleolar modulation for longevity attained by *xpo-1* silencing.

### Ribosomal protein RPL-11 is reduced by *xpo-1* silencing and regulates nucleolar size

**Fig. 4.**
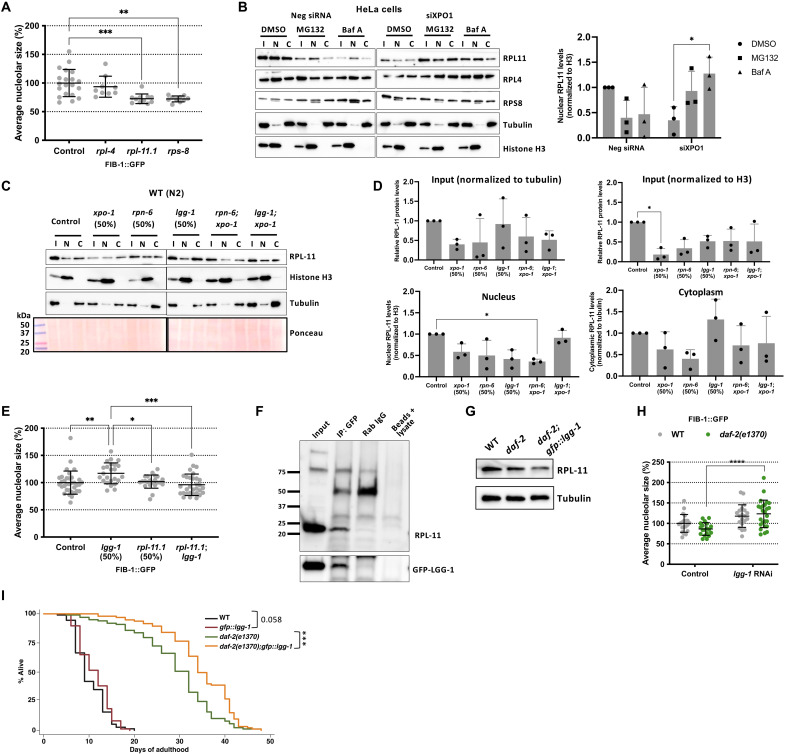
RPL-11 is regulated by XPO-1 and contributes to nucleolar size modulation. (**A**) Average nucleolar size (%) in FIB-1::GFP nematodes on ribosomal subunit gene RNAi versus control (*n* = 10 to 20 nematodes). (**B**) Representative blots for RPL11, RPL4, and RPS8 levels in input (I), nuclear (N), and cytoplasmic (C) fractions from HeLa cells upon *xpo1* or control RNAi and either proteasome (MG132) or lysosome (bafilomycin A1) inhibition. Graph represents RPL-11 levels in nuclear fractions for the same conditions as the blot. (**C**) Representative blot for RPL-11 protein levels in input (I), nuclear (N), and cytoplasmic (C) fractions from wild-type N2 nematodes on control and various RNAi for 96 hours. Histone H3 and tubulin were used as markers for nuclear and cytoplasmic fractions, respectively. (**D**) Densitometric quantification of blots in (C). Tubulin was used as reference for input and cytoplasm fractions, while histone H3 was used for input and nuclear fractions. (**E**) Epistatic analysis of *lgg-1* and *rpl-11.1* on average nucleolar size in FIB-1::GFP nematodes. (**F**) Representative coimmunoprecipitation (co-IP) of RPL-11 with GFP::LGG-1 using rabbit immunoglobulin G (IgG) and no antibody/sera as negative controls. Confirmation of IP by probing with GFP. (**G**) Representative blot of RPL-11 levels in long-lived *daf-2(e1370)* mutants with or without LGG-1 overexpression compared to wild-type N2. (**H**) Average nucleolar size in wild-type and *daf-2(e1370)* mutants in the presence and absence of *lgg-1* RNAi. (**I**) Life-span analysis of wild-type and *daf-2(e1370)* mutants with normal and overexpression of LGG-1. Data are represented as means ± SD. *P < 0.05, **P < 0.01, ***P < 0.001, and ****P < 0.0001 by Student’s *t* test or ANOVA or Mantel-Cox log rank test for life-span analyses. See table S1 for life-span statistics and repeats. See fig. S5E for *rpl-11.1* and *rpl-11.2* levels upon *rpl-11.1* silencing.

Ribosomal subunit proteins that are in stoichiometric excess have been known to accumulate around nucleoli when proteostasis is blocked (*35*). Since *xpo-1* RNAi led to a down-regulation of several ribosomal proteins, we sought to investigate whether this ribosomal subunit protein reduction along with enhanced proteostasis could contribute to smaller nucleoli. We selected three ribosomal subunit proteins (RPL-4, RPL-11.1, and RPS-8), all of which had reduced TE and were nuclear repartitioned with *xpo-1* RNAi (data files S2 and S3). Silencing each ribosomal subunit protein led to reduced nucleolar size (Fig. 4A). Since RPL11 is involved in p53-regulated nucleolar stress response (*36*) and RPL11 showed marked accumulation in nuclear fractions of *XPO1*-silenced HeLa cells upon blocking the proteasome and lysosome acidification with MG132 and bafilomycin A1, respectively (Fig. 4B), compared to that of RPL4 and RPS8, we hypothesized that this important ribosomal subunit may serve as a key target for nucleolar size regulation (*37*, *38*). We next investigated whether the proteostasis machinery in nematodes with silenced *xpo-1* contributes to RPL-11 degradation in the nucleus. Only *lgg-1* and not *rpn-6.1* RNAi caused RPL-11 to accumulate in nuclear fractions from nematodes with or without *xpo-1* silencing (Fig. 4, C and D, and fig. S4B), indicating the involvement of LGG-1 in RPL-11 degradation. Similarly, silencing the gene coding for lysosomal vacuolar-type adenosine triphosphatase, *vha-19*, caused RPL-11 to accumulate in nuclear fractions from nematodes with *xpo-1* silencing (fig. S5A), pointing toward a potential role for autophagy in nuclear RPL-11 turnover. Silencing *rpl-11.1* prevented nucleolar expansion induced by *lgg-1* RNAi (Fig. 4E), highlighting the contribution of RPL-11 to nucleolar size modulation by LGG-1. We also found RPL-11 to physically interact with LGG-1 by coimmunoprecipitation of RPL-11 using a GFP::LGG-1–expressing strain (Fig. 4F). Overexpressing LGG-1 in long-lived *daf-2* mutant nematodes further decreases RPL-11, supporting a role for LGG-1 in RPL-11 turnover (Fig. 4G). Silencing *lgg-1* reversed the phenotypes of small nucleoli and longevity in *daf-2* mutants (Fig. 4H and fig. S5B) (*39*), supporting a role for LGG-1 in nucleolar size modulation related to longevity. Overexpression of LGG-1 further extended life span of *daf-2* mutants and wild-type nematodes to a lesser extent (Fig. 4I). Modulating nucleolar size by silencing *rpl-11.1* in inducible muscle β-amyloid (Aβ)–expressing nematodes (*40*) significantly reduced the percentage of paralyzed worms after 48 hours of silencing, rescuing Aβ worms from paralysis (fig. S5C). Together, our data underscore the importance of RPL-11 and its degradation by LGG-1 in longevity.

## DISCUSSION

XPO-1/XPO1 is a conserved nuclear export receptor and is emerging as an important modulator of proteostasis and aging across phyla (*13*). Here, we combined transcriptomic and nucleocytoplasmic partitioning analyses to elucidate the mechanism by which XPO-1 modulates proteostasis and aging. Our transcriptomic analyses revealed pathways related to ribosome formation and function to be commonly diminished across several long-lived nematode models, highlighting them as convergent longevity mechanisms (*17*, *41*, *42*). In addition, our findings on repartitioning of ribosome subunits and translation initiation factors to the nucleus and subsequent translational attenuation upon *xpo-1* silencing concur with earlier reports of life-span extension with translational attenuation (*6*–*8*). The presence of translation initiation factors and other ribosome machinery inside the nucleus has been speculated to function in potential quality control for mRNA and ribosomes through nuclear translation (*43*, *44*). Although *xpo-1* RNAi suppressed global translation, categories involved in cellular maintenance and damage control such as neuronal function, stress response, transmembrane transport, and signaling were among the proteins with increased TE. Increase in ribosomal loading of specific mRNAs in the face of global translation reduction has been reported previously for mitochondrial genes during dietary restriction in *Drosophila* (*20*) and stress response genes during attenuation of translational initiation in *C. elegans* (*19*). We also found that genes with increased translation tended to have longer mRNAs, a pattern similar to earlier reports (*19*), indicative of a switch from growth encoding genes with shorter CDSs to somatic maintenance-related genes with longer CDSs. Translational up-regulation of certain categories such as proteostasis and energy metabolism has been observed despite a global decline in protein synthesis during aging (*45*), suggesting posttranscriptional mechanisms such as selective mRNA translation as important regulators of the aging proteome.

To investigate upstream mechanisms responsible for such translatomic shift, we focused on the nucleolus, the site for rRNA transcription and ribosome biogenesis (*22*). Silencing *xpo-1* caused nucleolar shrinkage stemming, in part, from down-regulation of nucleolar rRNA methyltransferase, fibrillarin (FIB-1/FBL), at transcript and protein levels. We also uncovered that nucleolar size is modulated by autophagic and proteasomal pathways, which coordinately control life span. Our findings mechanistically elucidate the reduction of nucleolar size and lower FIB-1 that characterize longevity models across phyla (*23*). Our data also suggest that dysregulated XPO-1 may lead to large nucleoli associated with diseases of aging such as Hutchinson-Gilford progeria (*46*), cancer (*47*), and oncogenic and replicative senescence (*48*). We found that increased FIB-1 cytoplasmic partitioning in *xpo-1*–silenced worms stemmed from a decrease in nuclear FIB-1, due to reduction in transcription and translation of FIB-1 mRNA. As p53 is a target of XPO1 (*49*, *50*), FIB-1 levels could have been altered via p53-mediated transcriptional repression. Silencing the worm p53 ortholog, *cep-1*, resulted in expanded nucleoli (fig. S5D). In addition, *xpo-1* silencing could also affect *c-myc* mRNA export and its subsequent translation by preventing export of eIF4E (IFE-5), which has an additional function as an mRNA export adaptor protein (*51*). Since *c-myc* is an *FBL* transcription stimulator (*52*), this could be an additional route for *xpo-1*–mediated *fib-1* transcriptional regulation. However, an ortholog of *c-myc* remains elusive in *C. elegans*. Reduction in FIB-1 levels decreases rRNA 2′-*O*-methylation and subsequently hampers the capacity of ribosomes to initiate translation from internal ribosome entry element sites and possibly regulate translational efficiency of specific mRNA for human ribosomes (*53*). Therefore, nucleolar modulation and FIB-1 reduction can affect rRNA processing, translation fidelity, and, ultimately, the translatome.

As nucleolar modulation appears to be a common theme across several longevity paradigms, including *xpo-1* inhibition, we explored whether proteostatic mechanisms, which are enhanced with longevity (*29*), could play a role in modulating nucleoli. Inhibiting autophagy or proteasomal degradation altered nucleolar size and suppressed longevity achieved by *xpo-1* inhibition, suggesting protein degradation capacity as a key driver of nucleolar dynamics in aging. Probing for possible targets of protein degradation pathways that influence nucleoli, we uncovered ribosomal large subunit protein, RPL-11, as a target of LGG-1–mediated degradation. RPL11 is known to contribute to nucleolar integrity by stabilizing p53 (*36*), and its degradation via LGG-1 in *xpo-1*–silenced worms could contribute to reduce nucleolar size. Although very little is known on the nuclear presence and roles of the LGG-1 ortholog, GABARAP, another better-characterized member of the autophagy-related protein 8 (ATG8) family, LC3, has been reported to have roles in the nucleus. A nuclear pool of LC3 is complexed with larger proteins, preventing its passive diffusion through the nuclear pore until autophagy is induced (*54*), and LC3 has been linked to nucleolar surveillance (*55*). Our observations on RPL-11 accumulation in nuclear fractions upon *lgg-1* silencing point toward degradation of RPL-11 via LGG-1. We report decreased RPL-11 levels and enhanced longevity of *daf-2* mutant worms overexpressing LGG-1, which underscores the involvement of RPL-11 as an LGG-1 target in longevity. In addition, *rpl-11.1* or *fib-1* silencing also partially rescued Aβ-expressing worms from paralysis, which underscores their importance as potential targets for neurodegeneration. Ribosomal protein mutations are also seen in ribosomopathies (*56*), diseases characterized by nucleolar and ribosomal defects, and one such disease, Diamond-Blackfan anemia, is accompanied by RPL11 mutations (*57*). Together, these findings demonstrate mechanistic cross-talk between the convergent longevity pathways of nucleolar modulation and autophagy.

**Fig. 5.**
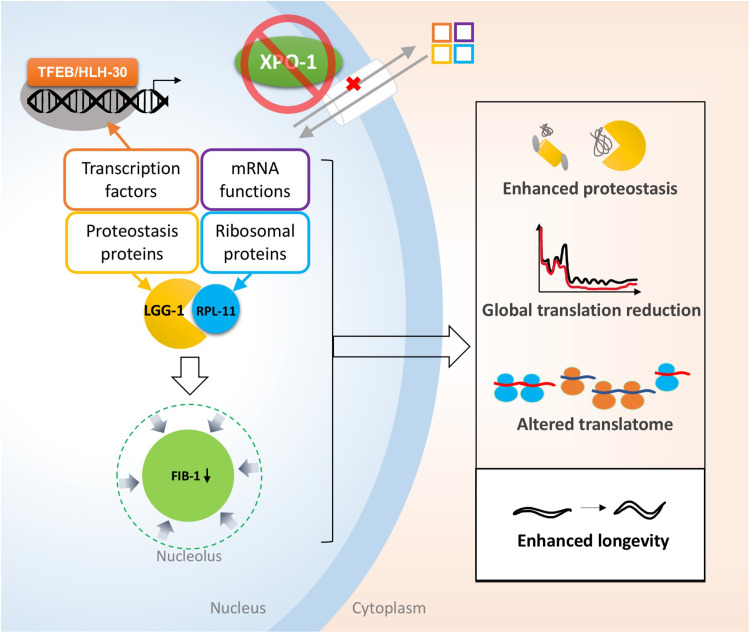
Coordination of proteostasis and ribosome dynamics regulates life span modulated by XPO-1. Inhibition of XPO-1 causes repartitioning of key proteins involved in mRNA functions, proteostasis, and ribosomal proteins to the nucleus. A concomitant reduction of nucleolar size is mediated by reduced levels of nucleolar protein FIB-1 and turnover of ribosomal subunit protein RPL-11 via its association with autophagy protein LGG-1. Protein redistribution and nucleolar constriction lead to enhanced proteostasis, global translation reduction, and altered translatome, ultimately enhancing longevity.

Blocking nuclear export by pharmacological inhibition of XPO1 has emerged as a therapeutic strategy in oncology, as elevated XPO1 levels are found in several cancers (*58*–*60*) and XPO1 inhibitors with clinical relevance have recently been found to improve sensitivity to anticancer therapies by inhibiting gene translation (*61*). Longevity, on the other hand, is accompanied by low XPO-1 levels, which differentially distributes proteins, and possibly mRNA, between the nucleus and cytoplasm, thus creating an environment that supports proteostatic resilience and cell survival (*62*). Here, we identified protein partitioning that supports prolongevity phenotypes, including nucleolar constriction, attenuation of ribosome biogenesis and protein synthesis, and enhanced proteostasis (Fig. 5). We also elucidated key players of *xpo-1*–modulated longevity, FIB-1 and RPL-11, both of which are critical determinants of nucleolar size. Last, we uncovered that LGG-1/GABARAP mediates the turnover of RPL-11, linking an autophagy protein to nucleolar size modulation. Together, our study reveals that nucleocytoplasmic partitioning of proteins is a key mechanism for longevity by influencing the specific degradation of ribosomal proteins with roles in nucleolar dynamics.

## MATERIALS AND METHODS

### Nematode strains and maintenance

*C. elegans* strains were maintained on nematode growth media agar plates seeded with *Escherichia coli* OP50 at 20°C and handled as described previously (*63*). Ethical approval for nematode experiments was not required by our institution. The list of strains used in this study is given in table S2.

### Cell culture

HeLa cells (American Type Culture Collection, CCL-2) were maintained in Dulbecco’s modified Eagle’s medium high glucose with 10% fetal bovine serum, 2 mM l-glutamine, and 1% penicillin-streptomycin at 37°C with 5% CO_2_ and 95% air.

### Method details

#### *Gene silencing in nematodes by RNAi*

Nematodes were synchronized by hypochlorite and raised at 20°C on OP50 *E. coli* bacteria until adulthood, after which they were transferred onto plates seeded with control bacteria (*E. coli* HT115 expressing vector L4440) or bacteria expressing double-stranded RNA (dsRNA) against the appropriate gene. Adult nematodes were separated from progeny by collection in M9 buffer and gravity sedimentation for 2 min and transferred onto fresh RNAi plates. Transfers were carried out each day until the final day of nematode collection. Nematodes were collected in M9 buffer and frozen until further use for RNA extraction of nucleocytoplasmic fractionation. The specificity of all bacterial RNAi clones used from the Ahringer library (*64*) for the appropriate dsRNA was confirmed by Sanger sequencing (Genewiz). Notably, *rpl-11.1* and *rpl-11.2* are duplicated gene pairs that are present on an autosome (chromosome V) and X chromosome, respectively. Therefore, the autosomal copy functions in germline development due to X chromosome inactivation (*65*). The RNAi sequences for *rpl-11.1* in the Ahringer library has about 90% identity with *rpl-11.2*, leading to silencing of *rpl-11.2* as well (fig. S5E). The antibody used in this study was raised using RPL11 peptide that has 81.66% identity to RPL11.1 and 82.25% to RPL-11.2.

### Gene silencing and proteostasis inhibition in HeLa cells

Gene silencing in HeLa cells was carried out by forward transfection by plating cells in culture dishes or on coverslips to ~60% confluence and then transfecting them using small interfering RNA (siRNA) to *XPO1* (s14937) or *GABARAP* (136382), respectively, for 48 hours using Lipofectamine RNAiMAX Transfection Reagent (Thermo Fisher Scientific) in Opti-MEM medium (Gibco). Thereafter, cells on coverslips were fixed using ice-cold methanol for nucleoli straining or were scraped off and used for protein extraction for Western blots. For studies on proteostasis inhibition, cells were treated for an additional 2 hours with 25 μM MG132 (Selleck Chemicals) to inhibit the proteasome or 4 hours with 400 nM bafilomycin A1 (Selleck Chemicals) to inhibit lysosome acidification. This was followed by staining or harvesting for protein collection as described above.

### Total RNA extraction and next-generation sequencing

Total RNA was extracted from approximately 3000 day 5 worms after 96 hours of RNAi using TRIzol reagent and further purified using QIAGEN RNeasy kit. cDNA was prepared as previously described (*14*). Library preparation and next-generation sequencing (NGS) was carried out by Genewiz using polyadenylate selection and Illumina HiSeq for biological replicates samples. Raw counts were gated to exclude genes with reads less than 10. Data analysis was carried out in iDEP.91 (*66*) using read counts (only ≥10) as the input and DESeq2 for calling differentially expressed genes (DEGs) and only significant DEGs (with false discovery rate <0.05 and fold change >1.5-fold). GO term analysis was carried out using WormCat (*16*).

### rRNA levels

Total RNA from control and RNAi-exposed worms at days 1, 10, and 20 of adulthood was extracted as described above. Bands of 26*S* and 18*S* rRNA were visualized on a Bioanalyzer 2100 chip (Agilent Technologies, Santa Clara, CA; Brown University Genomics Core). Gene expression levels for 47*S* pre-rRNA were measured in biological triplicates using iTaq Universal SYBR Green Supermix (Bio-Rad) and a Roche LightCycler 96 (Indianapolis, IN). As mature rRNAs (26*S* and 18*S*) and actin (*act-1*) were affected by *xpo-1* RNAi, we used *pmp-3* for normalization. Statistical analyses were performed using GraphPad Prism 7 (GraphPad Software). Primer sequences used are given in table S3.

### Nucleus-cytoplasm fractionation and proteomics

Following *xpo-1* RNAi or control for 96 hours from adulthood, adult worms were collected in M9 buffer and frozen at −80°C until further use. Frozen nematodes were resuspended in MSB buffer [10 mM tris-HCl and 250 mM sucrose (pH 7.4)] containing protease and phosphatase inhibitors (cOmplete Mini Protease Inhibitor Cocktail and PhosSTOP; Roche) and passed through a ball bearing homogenizer with 18-μm clearance for 40 passes. The lysate obtained was spun down at 500 rpm for 1 min to sediment cuticle and debris. Ten percent of the lysate was set aside as input sample. Nuclear fraction was pelleted by centrifugation at 3200 rpm for 10 min and resuspended in MSB buffer, while the supernatant, consisting of other organelles and cytoplasm, was set aside. Purity of the fractions was validated by Western blotting for tubulin (mostly cytoplasmic) and histone H3 (mostly nuclear). These fractions, along with input samples, were used for Western blot analysis or subjected to TMT labeling, followed by detection of peptides by mass spectrometry from biological duplicates (*67*). Briefly, trypsin was added to the samples (1:50, w/w) and incubated overnight at room temperature. A total of 50 μg of sample from each group was labeled with the TMTpro Kit (Thermo Scientific) on the basis of the quantification achieved from Qubit Fluorometric Quantitation (Thermo Scientific) [“Control, Nucleus” (126, 127N), “Control, Cytosol” (128 N, 128C), “*xpo-1*–silenced worm, Nucleus” (129C, 130N), and “*xpo-1*–silenced worm, Cytosol” (131N, 131C)]. The TMT-labeled peptides were mixed in equal ratios and analyzed simultaneously. The mixture was subsequently fractionated using high-pH reversed-phase fractionation (UltiMate 3000, Thermo Scientific). Thirty fractions were dissolved in buffer A (0.1% formic acid) and analyzed using a nanoflow liquid chromatography–tandem mass spectrometry system consisting of an Easy-nLC and a Orbitrap Exploris mass spectrometer (Thermo Scientific). The peptides were eluted using 90- to 100-min gradients from buffer B (95% acetonitrile and 0.1% formic acid) and introduced into the mass spectrometry instrument via nano-electrospray. Data analysis was carried out using a bioinformatics pipeline (*68*). Briefly, the raw datasets were processed for protein identification using the MS-GF+ (v9979, 08/05/2020) combined with the MS/MS automated selected ion chromatogram generator (MASIC) pipeline with a peptide mass tolerance of 20 parts per million, reporter ion mass/charge ratio tolerance half-width of 2 mDa, and a false discovery rate of 1% for proteins and peptides. All peak lists were searched against the UniProtKB/Swiss-Prot database (2020_09, 26,714 entries) of *C. elegans* sequences (taxonomy ID: 6239) with decoy using the parameters as follows: enzyme, trypsin; maximum missed cleavages, 2; fixed modification, carbamidomethylation (C), TMT tags (K, peptide N termini); and variable modifications, oxidation (M). Datasets with raw values were filtered to remove potential errors using the criteria as follows: elimination of contaminants and reversed sequences for each accession number. Protein relative expression values from the respective unique peptides (only in a single protein) were calculated by summing all peptide intensities of each protein and normalized to the number of the total intensity of each group to estimate the relative amounts of the different proteins within the fraction. (i) The total intensity of each protein (total) is the sum of the intensity of the protein in the nucleus (Nuc) and cytosol (Cyt). (ii) The proportion of the protein in the Nuc and Cyt was calculated according to the following calculation

Total=Nuc+Cyt (1)

$$\begin{array}{c} \text{Nuc}\%=\frac{\text{Nuc}}{\text{Total}}\times 100\\ \text{Cyt}\%=\frac{\text{Cyt}}{\text{Total}}\times 100 \end{array}$$ (2)

The resulting ratios were log-transformed (base = 2) to achieve a normal distribution, and then, log_2_ ratios were averaged per unique protein for subsequent analysis. All differentially expressed proteins were defined in the comparison of the Cyt and Nuc fractions using statistical methodology (multiple *t* test for adjusted *P* < 0.05 with the Holm-Sidak method).

### Polysome profiling and mRNA sequencing

Polysome extraction and profiling were carried out as described earlier (*19*). Briefly, wild-type nematodes fed on *xpo-1* RNAi or control bacteria for 96 hours were collected in ice-cold solubilization buffer [300 mM NaCl, 50 mM tris-HCl (pH 8.0), 10 mM MgCl_2_, 1 mM EGTA, 200 g of heparin/ml, 400 U of RNAsin/ml, 1 mM phenylmethylsulfonyl fluoride, 0.2 mg of cycloheximide/ml, and 1% Triton X-100] and homogenized for 1 min using a micropestle and incubated on ice for 15 min. Debris was separated by centrifuging at 20,000*g* for 10 min at 4°C. A fraction of the supernatant was set aside for total RNA extraction using TRIzol, while the rest was layered on top of a 10 to 50% sucrose gradient in high salt resolving buffer [140 mM NaCl, 25 mM tris-HCl (pH 8.0), and 10 mM MgCl_2_] at 38,000 rpm (>100,000*g*) for 1.5 hours at 4°C. Gradients were fractionated in a Biocomp fraction collector with simultaneous absorbance measurements at 252 nm to generate polysome profiles. RNA was extracted from biological duplicates from monosome and polysome fractions and together, with total RNA, was sequenced by NGS using Illumina HiSeq 2 × 150–base pair sequencing (Genewiz, South Plainfield, NJ). Differentially enriched transcripts were identified using DESeq2 and GO term enrichment by WormCat (*16*). TE was calculated as log_2_ [(polysome/monosome mRNA counts for *xpo-1* RNAi)/(polysome/monosome mRNA counts for control)] as described previously (*69*).

### Fluorescence microscopy and analysis

Nucleoli were visualized and imaged using the *fib-1::gfp* strain, unless mentioned otherwise. Adult nematodes were fed control or RNAi bacteria for 48 hours, and at least five nematodes per treatment were imaged by confocal microscopy using Olympus FV3000 inverted confocal laser scanning microscope (Leduc Bioimaging Facility, Brown University). *Z* stack reconstructions of images at ×10 magnification were used for nucleolar size quantification using particle analyzer tool in ImageJ. For HeLa cells, nucleoli were visualized using SYTO RNASelect dye (Invitrogen, Carlsbad, CA) that preferentially stains RNA as per the manufacturer’s protocol, followed by epifluorescence microscopy using a Zeiss Axiovert 200M fluorescence microscope. Images were analyzed for nucleolar size using particle analyzer in ImageJ from three independent experiments using at least 10 worms, unless mentioned otherwise.

### Immunoprecipitation

The *gfp::lgg-1* strain was grown to adulthood, and day 1 worms were either used for immunoprecipitation (IP) or plated on appropriate RNAi plates for 72 hours and then collected for IP. Worms were collected in IP buffer (137 mM NaCl, 2.7 mM KCl, 4.3 mM Na_2_HPO_4_, 1.47 mM KH_2_PO_4_, 250 mM sucrose, and protease inhibitor) and homogenized for 40 passes through a ball bearing homogenizer. The lysate was centrifuged at 500 rpm for 1 min at 4°C to remove debris. From the supernatant, 10% was set aside as input, and the remaining supernatant was used for IP for GFP or rabbit immunoglobulin G (IgG) or no-antibody control. The lysate was rotated with the appropriate antibody or IgG control overnight at 4°C, followed by incubation with SureBeads magnetic beads (Bio-Rad, Hercules, CA) overnight at 4°C. Beads were magnetically separated followed by five washes as per the manufacturer’s protocol and eluted in Laemmli buffer containing 355 mM β-mercaptoethanol and 50 mM dithiothreitol at 70°C for 10 min. Immunoprecipitated material was separated and visualized by Western blotting. Antibody details for IP and Western blotting are given in table S4.

### Western blotting

Protein extraction from whole worms or cells was carried out by homogenization in radioimmunoprecipitation assay buffer (50 mM tris-HCl, 150 mM NaCl, 1 mM EDTA, 2% SDS, and protease inhibitor) using a micropestle followed by rotation for 1 hour at 4°C and centrifugation at 13,000 rpm for 20 min. Protein concentration was estimated using DC protein assay kit (Bio-Rad). Western blotting was carried out from whole worm or cell lysates or nuclear and non-nuclear fraction from worms or immunoprecipitated material as described before (*13*). Briefly, 20 μg of proteins were loaded onto 4 to 15% TGX gels (Bio-Rad) and resolved by electrophoresis. Proteins were transferred using the Trans-Blot Turbo Transfer System (Bio-Rad) onto nitrocellulose membrane (Bio-Rad). Immunoblotting was conducted using appropriate antibodies, and proteins were visualized with ECL reagents (SuperSignal West Femto, Pierce) using a ChemiDoc Imaging System (Bio-Rad).

### Survival assays

Nematodes were synchronized by bleaching and grown to adulthood on OP50 *E. coli*. On reaching adulthood, they were plated on control or RNAi plates and were scored for survival throughout their life span. They were transferred onto fresh plates until the postreproductive stage.

### Paralysis assays

Inducible Aβ1-42–expressing nematodes were grown until the L4 stage at 20°C, after which they were transferred to RNAi or control plates at 25°C to induce Aβ expression in three independent experiments. They were scored for paralysis and death every day until all worms on the plates were paralyzed.

### Statistical methods

Student’s *t* test was used to compare a single variable between two conditions. One-way analysis of variance (ANOVA) was used to compare a single parameter between three or more conditions, and two-way ANOVA was used to compare multiple parameters. In ANOVAs, Tukey’s multiple comparisons test was used, and all conditions were compared to all other conditions. Statistical analyses for these were performed using the software GraphPad Prism 9. Mantel-Cox log rank test was used for life-span analyses and was performed using Stata SE 15.0.

## Supplementary Materials

### This PDF file includes:

Figs. S1 to S5 Tables S1 to S4 References

## Notes

View/request a protocol for this paper from *Bio-protocol*.
