# Supplementary material for: Exportin 1 modulates life span by regulating nucleolar dynamics via the autophagy protein LGG-1/GABARAP
Source: Sci Adv. 2022 Apr 1;8(13):eabj1604. doi: 10.1126/sciadv.abj1604 (PMC10938577; doi:10.1126/sciadv.abj1604)
Supplement: Supplementary file 1 — Figs. S1 to S5 Tables S1 to S4 References [file sciadv.abj1604_sm.pdf]

Science Advances
AAAS

Supplementary Materials for

Exportin 1 modulates life span by regulating nucleolar dynamics via the autophagy protein LGG-1/GABARAP

Anita V. Kumar, Taewook Kang, Tara G. Thakurta, Celeste Ng, Aric N. Rogers, Martin R. Larsen, Louis R. Lapierre*

*Corresponding author. Email: louis_lapierre@brown.edu

Published 1 April 2022, *Sci. Adv.* **8**, eabj1604 (2022)
DOI: 10.1126/sciadv.abj1604

The PDF file includes:

Figs. S1 to S5
Tables S1 to S4
Legends for data files S1 to S4
References

Other Supplementary Material for this manuscript includes the following:

Data files S1 to S4

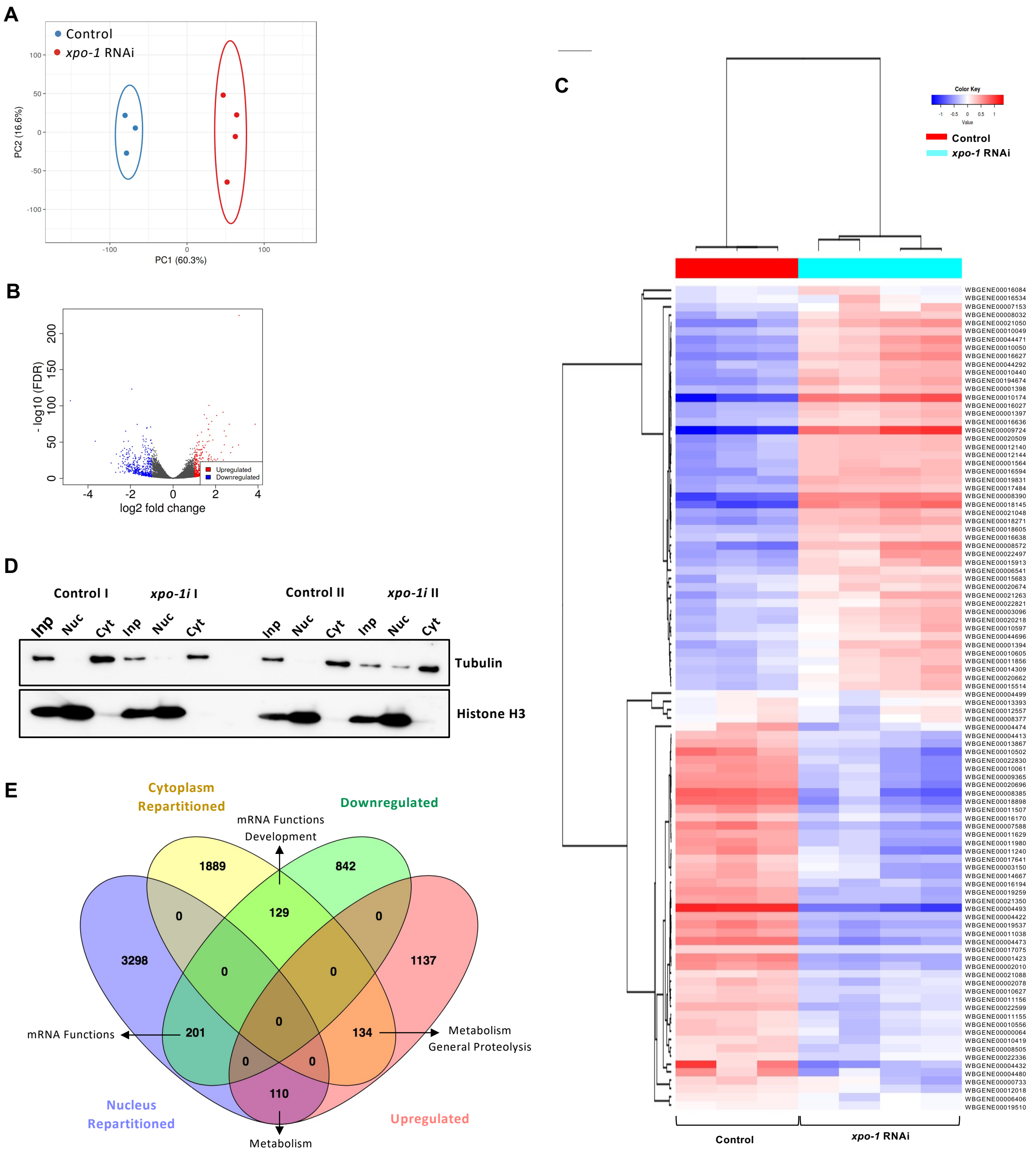

Fig. S1. Transcriptomic and subcellular proteomic analyses of *xpo-1* inhibited nematodes

(A) Principal component analysis for biological replicate samples used for RNA-seq prepared using ClustaVis tool. (B) Volcano plot depicting significantly up-and down-regulated genes in *xpo-1* RNAi compared to control prepared in iDEP .91. (C) Heatmap of top 100 differentially regulated genes obtained in *xpo-1* RNAi compared to control prepared using iDEP .91. (D) Histone H3 and Tubulin as markers for nuclear and non-nuclear (cytoplasmic) fractions, respectively, of input (Inp), nucleus (Nuc), and cytoplasm (Cyt) samples used for TMT-MS proteomics. (E) Venn diagram of overlapping hits from transcriptomics and proteomics datasets prepared using Venny 2.1 depicting enriched GO categories obtained using WormCat.

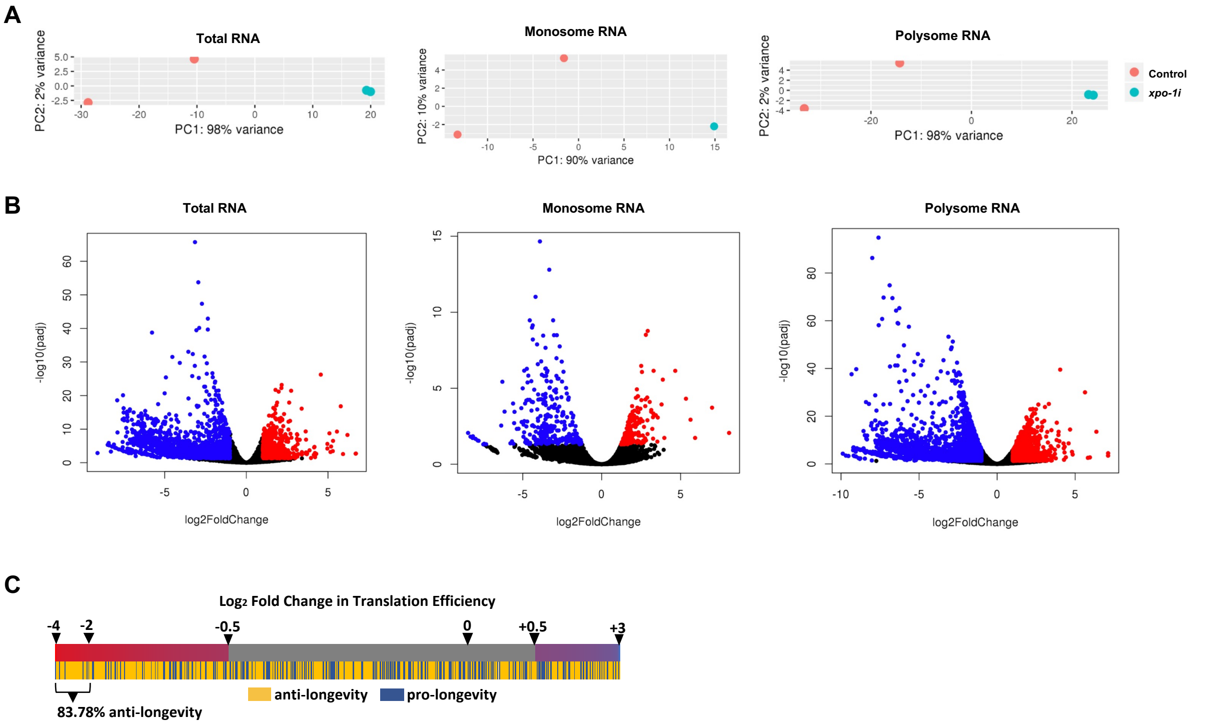

Fig. S2. mRNA sequencing analysis from total, monosome, and polysome fractions from *xpo-1* inhibited animals

(A) Principal component analyses for total, monosome, and polysome RNA samples used for RNA-seq. (B) Volcano plots representing up- and down-regulated genes obtained in total, monosome, and polysome RNA from *xpo-1* RNAi samples compared to control. (C) Heatmap representation of proteins with altered translation efficiency that also have a known role in longevity as per the GenAge database. The percentage of anti-longevity genes identified in our dataset of highly decreased log2 TE is indicated.

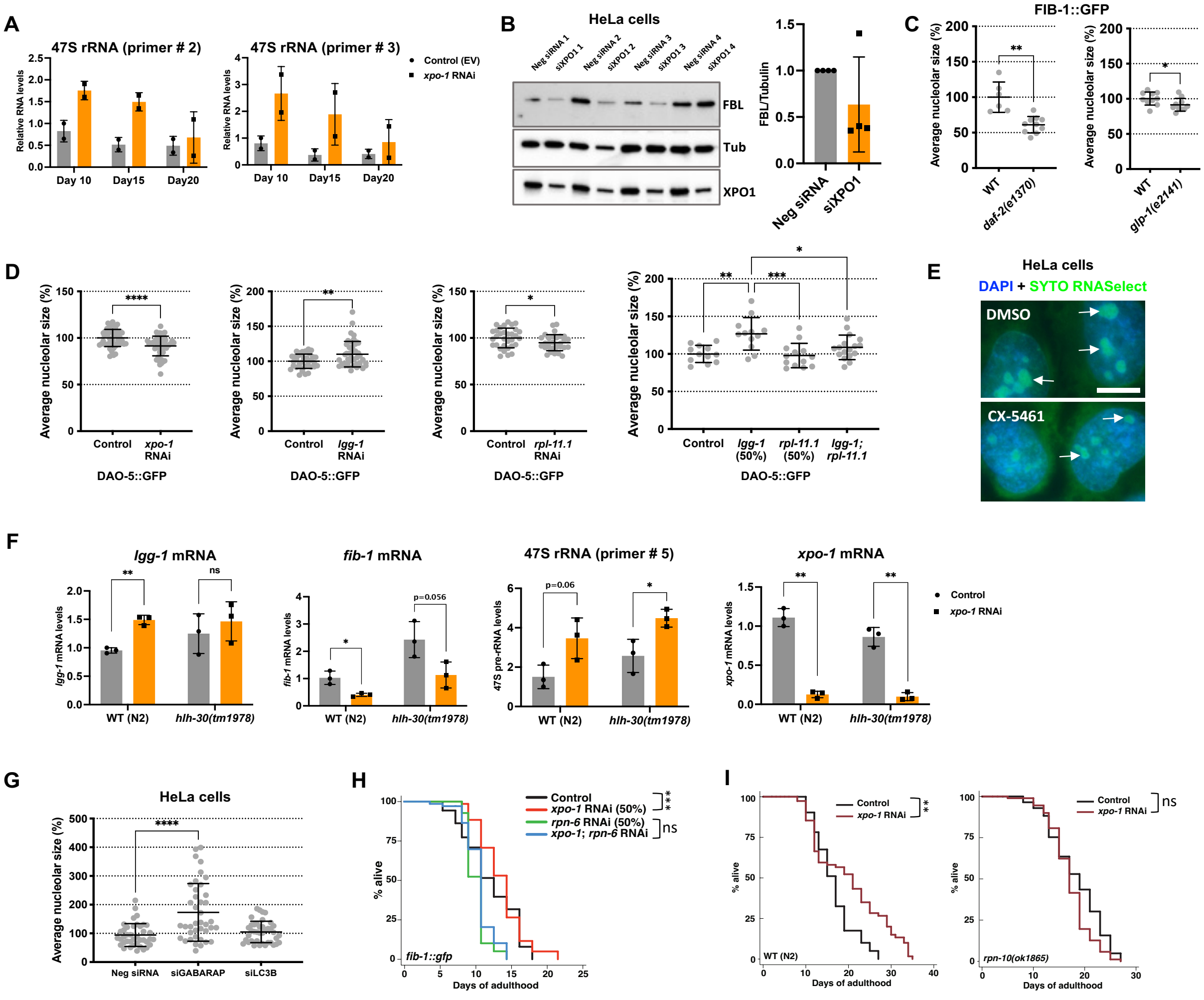

Fig. S3. XPO-1 regulates nucleolar size

(A) Expression of 47S pre-rRNA transcripts by qPCR in *xpo-1* RNAi compared to control using additional primers as confirmation (biological duplicates). See Supplementary table S6 for primer sequences. (B) FBL protein levels in HeLa cells treated with negative control or *xpo1* siRNA. Graph represents densitometry of the blot using Tubulin as the reference. (C) Average nucleolar size in *daf-2(e1370)* and *glp-1(e2141)* mutants expressing FIB-1::GFP. Nucleolar size was quantified on Day 1 of adulthood in *daf-2(e1370)* mutants raised at 15°C and *glp-1(e2141)* mutants raised at 25°C. (D) Average nucleolar size in nematodes expressing DAO-5::GFP subjected to *xpo-1, lgg-1, rpl-11.1*, or a combination of *lgg-1* and *rpl-11.1* RNAi for 48h from adulthood. (E) Nucleoli (arrows) stained using SYTO RNASelect in HeLa cells treated with RNA Polymerase I inhibitor, CX-5461 (1µM) or vehicle control (DMSO). Scale bar = 50µm. (F) RNA levels of *lgg-1, fib-1*, 47S rRNA, and *xpo-1* measured by qPCR in N2 wildtype and *hlh-30(tm1978)* null mutants upon control (L4440) or *xpo-1* RNAi from adulthood for 72h (biological triplicates). See Supplementary table S6 for primer sequences. (G) Average nucleolar size in HeLa cells treated with negative siRNA or siRNA to *gabarap* or *lc3* for 48h. (H) Lifespan analyses of *fib-1::gfp* nematodes on *xpo-1* and *rpn-6.1* RNAi alone or in combination compared to control. (I) Lifespan analyses of WT (N2) or *rpn-10(ok1865)* mutants on control or *xpo-1* RNAi from adulthood. *p<0.05, **p<0.01, ***p<0.001, ****p<0.0001, ns=not significant by Student's t test or ANOVA or Mantel-Cox log rank test for lifespan analyses. See Table S1 for lifespan statistics and repeats.

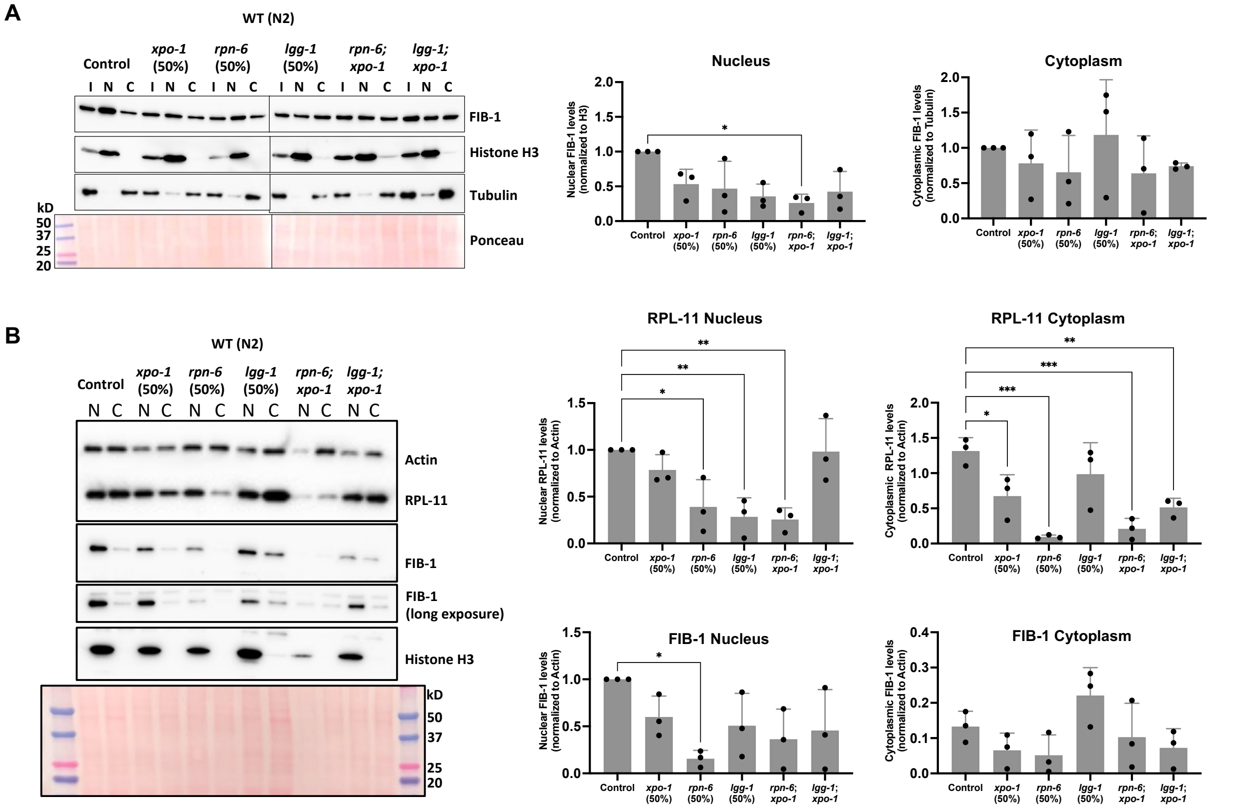

Fig. S4. XPO-1 regulates ribosomal protein levels in an LGG-1-dependent manner

(A) Representative blot showing FIB-1 protein levels in nuclear and cytoplasmic fractions of wildtype N2 worms upon various RNAi for 72h. Graphs represent densitometric quantification of FIB-1 in nuclear and cytoplasmic fractions using Histone H3 and Tubulin as references, respectively. (B) Representative blot showing RPL-11 and FIB-1 levels in nuclear and cytoplasmic fractions of wildtype N2 worms upon various RNAi for 72h. Graphs represent densitometric quantification of RPL-11 and FIB-1 levels in nuclear and cytoplasmic fractions normalized to Actin. Data are represented as mean ± SD from three independent trials. I = Input, N = Nuclear, C = Cytoplasmic fractions, respectively. *$p<0.05$, **$p<0.01$, ***$p<0.001$, ****$p<0.0001$ by one-way ANOVA

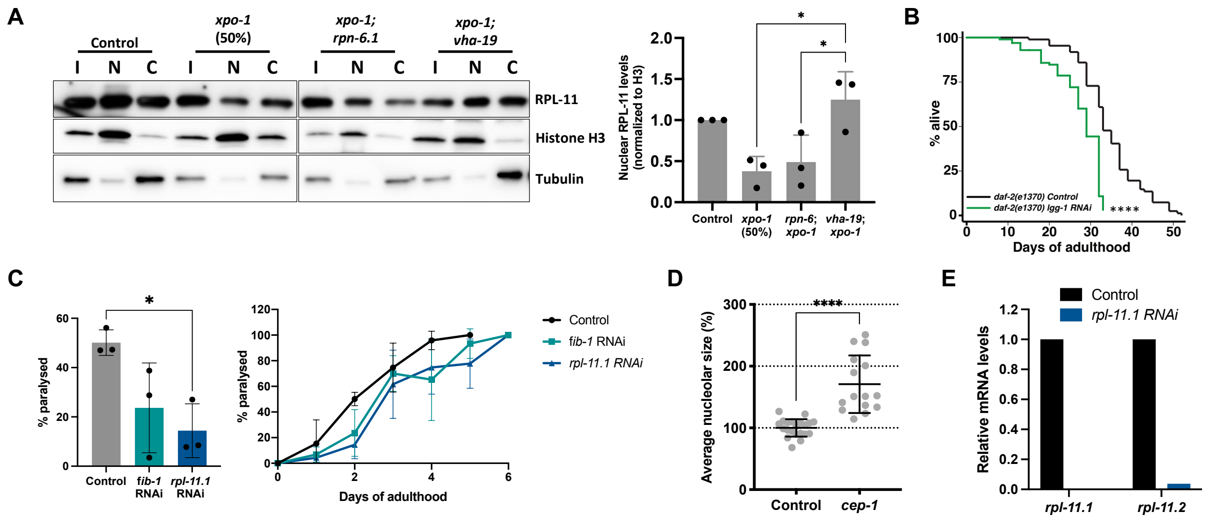

Fig. S5. Lifespan, nucleolar size and proteostasis measurements

(A) Representative blot for RPL-11 protein levels in input (I), nuclear (N), and cytoplasmic (C) fractions from wildtype N2 nematodes on control and various RNAi for 96h. Histone H3 and Tubulin were used as markers for nuclear and cytoplasmic fractions, respectively. Graph represents densitometric quantification of blots in nuclear fractions (normalized to Histone H3). (B) Lifespan analysis of *daf-2(e1370)* mutants upon *lgg-1* RNAi compared to control. (C) Paralysis assay in nematodes expressing heat-inducible Aβ in muscles with *fib-1* and *rpl-11.1* RNAi compared to control. Graphs represent percent paralyzed worms after 48h or RNAi and through the entire time course. (D) Nucleolar size upon *cep-1* RNAi for 48h compared to control. Data are represented as mean ± SD from three independent trials. (E) *rpl-11.1* and *rpl-11.2* mRNA levels upon *rpl-11.1* RNAi compared to control (technical triplicates). Data are represented as mean ± SD. *$p<0.05$, **$p<0.01$, ***$p<0.001$, ****$p<0.0001$ by Student's *t* test or ANOVA or Mantel-Cox log rank test for lifespan analyses. See Table S1 for lifespan statistics and repeats.

Strain	Conditions	Events Observed/N	Mean Lifespan	Maximum Lifespan	% Difference	P value	
N2	L4440	66/100	19.84	34			
	xpo-1 (50%)	76/100	21.35	30	7.61	0.26	
	rpn-6.1 (50%)	82/100	13.46	16	-32.16	<0.001	
	rpn-6;xpo-1	83/100	13.75	18	-30.70	<0.001	
	lgg-1 (50%)	71/100	16.74	24	-15.63	<0.001	
	lgg-1,xpo-1	88/100	17.92	26	-9.68	0.0072	
N2	L4440	82/100	21.49	35			
	xpo-1 (50%)	73/100	23.27	35	8.28	0.0231	
	rpn-6.1 (50%)	81/100	14.89	19	-30.71	<0.001	
	rpn-6;xpo-1	87/100	14.52	17	-32.43	<0.001	
	lgg-1 (50%)	58/100	21.49	31	0.00	0.7176	
	lgg-1,xpo-1	84/100	17.32	23	-19.40	<0.001	
N2	L4440	44/100	14.51	25			
	xpo-1 (50%)	46/100	21	29	44.73	<0.001	
	rpn-6.1 (50%)	69/100	11.75	17	-19.02	<0.001	
	rpn-6;xpo-1	83/100	11.42	19	-21.30	<0.001	
	lgg-1 (50%)	64/100	11.37	21	-21.64	<0.001	
	lgg-1,xpo-1	70/100	17.81	23	22.74	<0.001	
pfib-1::fib-1::gfp	L4440	37/80	13.72	26			
	xpo-1	44/80	19.48	32	41.98	0.0001	
pfib-1::fib-1::gfp	L4440	47/100	15.84	29			
	xpo-1	44/100	18.45	32	16.48	0.0327	
pfib-1::fib-1::gfp	L4440	36/100	12.95	25			
	xpo-1	37/100	22.42	39	73.13	<0.001	
pfib-1::fib-1::gfp	L4440	30/100	13.55	20			
	xpo-1	59/100	15.21	28	12.25	0.1768	
pfib-1::fib-1::gfp	L4440	47/100	15.84	29			
	xpo-1 (50%)	49/100	21.61	40	36.43	0.0001	

	rpn-6.1 (50%)	59/100	13.2	19	-16.67	<0.0001	
	rpn-6;xpo-1	61/100	13.63	17	-13.95	<0.0001	
pfib-1::fib-1::gfp	L4440	36/100	12.95	25			
	xpo-1 (50%)	34/100	20.32	39	56.91	<0.001	
	rpn-6.1 (50%)	45/100	13.39	18	3.40	0.662	
	rpn-6;xpo-1	48/100	13.75	18	6.18	0.1883	
ncl-1(e1942);pfib-1::fib-1::gfp	L4440	48/100	15.06	28			
	xpo-1	65/100	12.52	25	-16.87	0.0016	
ncl-1(e1942);pfib-1::fib-1::gfp	L4440	51/100	14.87	27			
	xpo-1	64/100	12.96	27	-12.84	0.0194	
ncl-1(e1942);pfib-1::fib-1::gfp	L4440	50/100	17.97	26			
	xpo-1	67/100	13.79	20	-23.26	<0.001	
N2	25°C	78/100	10.33618	21			
plgg-1::gfp::lgg-1	25°C	85/100	10.47973	20	1.39	0.5635	with respect to N2
daf-2(e1370)	25°C	92/100	29.43	55			
daf-2(e1370);plgg-1::gfp::lgg-1	25°C	93/100	35.36	55	20.15	<0.001	with respect to *daf-2 (e1370)*
N2	25°C	84/100	10.18	20			
plgg-1::gfp::lgg-1	25°C	95/100	11.25	19	10.51	0.058	with respect to N2
daf-2(e1370)	25°C	84/100	28.37	47			
daf-2(e1370);plgg-1::gfp::lgg-1	25°C	96/100	39.3	55	38.53	<0.001	with respect to *daf-2 (e1370)*
N2	25°C	79/100	10.89	14			
plgg-1::gfp::lgg-1	25°C	88/100	11.85	21.00	8.82	0.0019	with respect to N2
daf-2(e1370)	25°C	86/100	25.11	46			
daf-2(e1370);plgg-1::gfp::lgg-1	25°C	92/100	31.49	46	25.41	<0.001	with respect to *daf-2 (e1370)*
daf-2(e1370)	25°C	83/100	34.7	52			
	25°C, *lgg-1*	80/100	27.69	33	-20.20	<0.0001	

daf-2(e1370)	25°C	29/80	35.01968	57			
	25°C, *lgg-1*	64/80	32.28013	43	-7.82	0.0561	
daf-2(e1370)	25°C	87/100	23.7	40			
	25°C, *lgg-1*	83/100	27.35	34	15.40	0.0025	
N2	L4440	65/100	16.41	26			
	xpo-1	77/100	18.6	27	13.34	0.0026	
N2	L4440	49/100	16.61	27			
	xpo-1	67/100	20.24	35	21.8543046	0.0043	
rpn-10(ok1865)	L4440	77/100	16.84	22			
	xpo-1	87/100	16.12	24	-4.27	0.4894	
rpn-10(ok1865)	L4440	84/100	18.39	27			
	xpo-1	87/100	17.21	27	-6.4165307	0.0173	

Table S1. Details of survival analyses performed in the study

**Strains used in this study**	
N2	Wild-type (Kenyon lab - CF)
LRL103	ptnIs001 [*Pfib-1*::*fib-1*::*gfp* fosmid::cbunc-119(+)]; *unc-119(ed3)*] (Ref. 25) outcrossed with N2 3 times
CF2218	*ncl-1(e1942) III*
LRL78	*ncl-1(e1942) III;*ptnIs001 [*Pfib-1*::*fib-1*::*gfp* fosmid::cbunc-119(+)]; *unc-119(ed3)*]
CF1041	*daf-2(e1370) III*
MAH14	*daf-2(e1370) III;*adIs2122 [*lgg-1*::*gfp* + *rol-6(su1006)*]
DA2123	adIs2122 [*lgg-1p*::*gfp*::*lgg-1* + *rol-6(su1006)*]
LRL41	GMC101 (dvIs100 [*unc-54p*::A-beta-1-42::*unc-54* 3'-UTR + *mtl-2p*::*gfp*] outcrossed with N2 4 times
LRL31	FX1978 [*hlh-30 (tm1978)]* outcrossed with N2 4 times
VC1369	*rpn-10(ok1865)*
CPB089	*ptnsIs050 (dao-5::gfp)*

Table S2. List of *C. elegans* strains used in the study

Gene	Primer Sequence	Reference
47S pre rRNA (F) #5	CTTCACGGACATGCGGTGAT	70
47S pre rRNA (R) #5	TGTGATGCTTCTGGACTAGG	
47S pre rRNA (F) #2	GATCCATAGATATTGCTGATGATTC	24
47S pre rRNA (R) #2	CGCAGACATATAGTCTAGCGAG	
47S pre rRNA (F) #3	AATACTGGGATTCGTCTA	
47S pre rRNA (R) #3	GAGTTCAGGTTGAGATTAG	
fib-1 (F)	TGCTTGTCGGAATGGTCGAT	
fib-1 (R)	TCTCTCATACGGCTCCAGGG	
lgg-1 (F)	ACCCAGACCGTATTCCAGTG	14
lgg-1 (R)	ACGAAGTTGGATGCGTTTTC	
pmp-3 (F)	GTTCCCGTGTTCATCACTCAT	
pmp-3 (R)	ACACCGTCGAGAAGCTGTAGA	
rpl-11.1 (F)	ACGGACGTCGAAAAACAGAC	
rpl-11.1 (R)	GCTCGAGCACCTTAGCG	
rpl-11.2 (F)	GATTCGGAGTCCAGGAGCAC	
rpl-11.2 (R)	TCTTCTCTTGGCGATACGGC	

Table S3. List of qPCR primers used in the study

Antibodies	Application	Source
Fibrillarin	Western blot	Novus Bio. NB300-269
RPL11	Western blot	Proteintech 16277-1-AP
Histone H3	Western blot	Abcam ab1791
Tubulin	Western blot	Abcam ab6160
XPO1	Western blot	Santa Cruz Biotechnology sc-74454
GFP	Western blot	Santa Cruz Biotechnology sc-9996
GFP	Immunoprecipitation	Proteintech 50430-2-AP
IgG Control	Immunoprecipitation	Proteintech 30000-0-AP
HRP Goat Anti-Mouse	Western blot	Li-Cor 926-80010
HRP Goat Anti-Rabbit	Western blot	Li-Cor 926-80011
HRP Goat Anti-Rat	Western blot	Life Technologies 31470
VeriBlot IP Detection Reagent HRP	Western blot	Abcam ab131366

Table S4. List of antibodies used in the study

Data S1. (separate file)

List of all differentially regulated transcripts with false discovery rate <0.05 and fold change >1.5 fold obtained by mRNA sequencing from *xpo-1* RNAi nematodes compared to control

Data S2. (separate file)

List of all nuclear and cytoplasmic repartitioned proteins upon *xpo-1* RNAi compared to control identified using TMT-MS analysis and repartitioned proteins that were also earlier found to be XPO1 cargo (15)

Data S3. (separate file)

List of all transcripts with altered translation efficiency upon *xpo-1* RNAi compared to control

Data S4. (separate file)

List of all transcripts uniquely altered only in polysome fractions upon *xpo-1* RNAi compared to control

REFERENCES AND NOTES

1. A. Komeili, E. K. O'Shea, New perspectives on nuclear transport. *Annu. Rev. Genet.* **35**, 341–364 (2001).

2. T. Guttler, D. Gorlich, Ran-dependent nuclear export mediators: A structural perspective. *EMBO J.* **30**, 3457–3474 (2011).

3. D. Xu, A. Farmer, G. Collett, N. V. Grishin, Y. M. Chook, Sequence and structural analyses of nuclear export signals in the NESdb database. *Mol. Biol. Cell* **23**, 3677–3693 (2012).

4. K. Thakar, S. Karaca, S. A. Port, H. Urlaub, R. H. Kehlenbach, Identification of CRM1-dependent nuclear export cargos using quantitative mass spectrometry. *Mol. Cell. Proteomics* **12**, 664–678 (2013).

5. S. Francisco, M. Ferreira, G. Moura, A. R. Soares, M. A. S. Santos, Does proteostasis get lost in translation? Implications for protein aggregation across the lifespan. *Ageing Res. Rev.* **62**, 101119 (2020).

6. A. Chiocchetti, J. Zhou, H. Zhu, T. Karl, O. Haubenreisser, M. Rinnerthaler, G. Heeren, K. Oender, J. Bauer, H. Hintner, M. Breitenbach, L. Breitenbach-Koller, Ribosomal proteins Rpl10 and Rps6 are potent regulators of yeast replicative life span. *Exp. Gerontol.* **42**, 275–286 (2007).

7. M. Hansen, S. Taubert, D. Crawford, N. Libina, S. J. Lee, C. Kenyon, Lifespan extension by conditions that inhibit translation in *Caenorhabditis elegans*. *Aging Cell* **6**, 95–110 (2007).

8. K. Z. Pan, J. E. Palter, A. N. Rogers, A. Olsen, D. Chen, G. J. Lithgow, P. Kapahi, Inhibition of mRNA translation extends lifespan in *Caenorhabditis elegans*. *Aging Cell* **6**, 111–119 (2007).

9. A. R. Hipkiss, On why decreasing protein synthesis can increase lifespan. *Mech. Ageing Dev.* **128**, 412–414 (2007).

10. T. von der Haar, J. E. Leadsham, A. Sauvadet, D. Tarrant, I. S. Adam, K. Saromi, P. Laun, M. Rinnerthaler, H. Breitenbach-Koller, M. Breitenbach, M. F. Tuite, C. W. Gourlay, The control of translational accuracy is a determinant of healthy ageing in yeast. *Open Biol.* **7**, 160291 (2017).

11. J. Xie, V. de Souza Alves, T. von der Haar, L. O'Keefe, R. V. Lenchine, K. B. Jensen, R. Liu, M. J. Coldwell, X. Wang, C. G. Proud, Regulation of the elongation phase of protein synthesis enhances translation accuracy and modulates lifespan. *Curr. Biol.* **29**, 737–749.e5 (2019).

12. K. K. Steffen, A. Dillin, A ribosomal perspective on proteostasis and aging. *Cell Metab.* **23**, 1004–1012 (2016).

13. M. J. Silvestrini, J. R. Johnson, A. V. Kumar, T. G. Thakurta, K. Blais, Z. A. Neill, S. W. Marion, V. St Amand, R. A. Reenan, L. R. Lapierre, Nuclear export inhibition enhances HLH-30/TFEB activity, autophagy, and lifespan. *Cell Rep.* **23**, 1915–1921 (2018).

14. L. R. Lapierre, C. D. De Magalhaes Filho, P. R. McQuary, C. C. Chu, O. Visvikis, J. T. Chang, S. Gelino, B. Ong, A. E. Davis, J. E. Irazoqui, A. Dillin, M. Hansen, The TFEB orthologue HLH-30 regulates autophagy and modulates longevity in *Caenorhabditis elegans*. *Nat. Commun.* **4**, 2267 (2013).

15. K. Kirli, S. Karaca, H. J. Dehne, M. Samwer, K. T. Pan, C. Lenz, H. Urlaub, D. Gorlich, A deep proteomics perspective on CRM1-mediated nuclear export and nucleocytoplasmic partitioning. *eLife* **4**, (2015).

16. A. D. Holdorf, D. P. Higgins, A. C. Hart, P. R. Boag, G. J. Pazour, A. J. M. Walhout, A. K. Walker, WormCat: An online tool for annotation and visualization of *Caenorhabditis elegans* genome-scale data. *Genetics* **214**, 279–294 (2020).

17. M. M. Senchuk, D. J. Dues, C. E. Schaar, B. K. Johnson, Z. B. Madaj, M. J. Bowman, M. E. Winn, J. M. Van Raamsdonk, Activation of DAF-16/FOXO by reactive oxygen species contributes to longevity in long-lived mitochondrial mutants in *Caenorhabditis elegans*. *PLOS Genet.* **14**, e1007268 (2018).

18. B. N. Heestand, Y. Shen, W. Liu, D. B. Magner, N. Storm, C. Meharg, B. Habermann, A. Antebi, Dietary restriction induced longevity is mediated by nuclear receptor NHR-62 in *Caenorhabditis elegans*. *PLOS Genet.* **9**, e1003651 (2013).

19. A. N. Rogers, D. Chen, G. McColl, G. Czerwieniec, K. Felkey, B. W. Gibson, A. Hubbard, S. Melov, G. J. Lithgow, P. Kapahi, Life span extension via eIF4G inhibition is mediated by posttranscriptional remodeling of stress response gene expression in *C. elegans*. *Cell Metab.* **14**, 55–66 (2011).

20. B. M. Zid, A. N. Rogers, S. D. Katewa, M. A. Vargas, M. C. Kolipinski, T. A. Lu, S. Benzer, P. Kapahi, 4E-BP extends lifespan upon dietary restriction by enhancing mitochondrial activity in Drosophila. *Cell* **139**, 149–160 (2009).

21. R. Tacutu, D. Thornton, E. Johnson, A. Budovsky, D. Barardo, T. Craig, E. Diana, G. Lehmann, D. Toren, J. Wang, V. E. Fraifeld, J. P. de Magalhaes, Human ageing genomic resources: New and updated databases. *Nucleic Acids Res.* **46**, D1083–D1090 (2018).

22. I. Grummt, The nucleolus-guardian of cellular homeostasis and genome integrity. *Chromosoma* **122**, 487–497 (2013).

23. V. Tiku, C. Jain, Y. Raz, S. Nakamura, B. Heestand, W. Liu, M. Spath, H. E. D. Suchiman, R. U. Muller, P. E. Slagboom, L. Partridge, A. Antebi, Small nucleoli are a cellular hallmark of longevity. *Nat. Commun.* **8**, 16083 (2017).

24. V. Tiku, C. Kew, P. Mehrotra, R. Ganesan, N. Robinson, A. Antebi, Nucleolar fibrillarin is an evolutionarily conserved regulator of bacterial pathogen resistance. *Nat. Commun.* **9**, 3607 (2018).

25. D. Tollervey, H. Lehtonen, R. Jansen, H. Kern, E. C. Hurt, Temperature-sensitive mutations demonstrate roles for yeast fibrillarin in pre-rRNA processing, pre-rRNA methylation, and ribosome assembly. *Cell* **72**, 443–457 (1993).

26. S. C. Weber, C. P. Brangwynne, Inverse size scaling of the nucleolus by a concentration-dependent phase transition. *Curr. Biol.* **25**, 641–646 (2015).

27. J. Berry, S. C. Weber, N. Vaidya, M. Haataja, C. P. Brangwynne, RNA transcription modulates phase transition-driven nuclear body assembly. *Proc. Natl. Acad. Sci. U.S.A.* **112**, E5237–E5245 (2015).

28. D. Drygin, A. Lin, J. Bliesath, C. B. Ho, S. E. O'Brien, C. Proffitt, M. Omori, M. Haddach, M. K. Schwaebe, A. Siddiqui-Jain, N. Streiner, J. E. Quin, E. Sanij, M. J. Bywater, R. D. Hannan, D. Ryckman, K. Anderes, W. G. Rice, Targeting RNA polymerase I with an oral small molecule CX-5461 inhibits ribosomal RNA synthesis and solid tumor growth. *Cancer Res.* **71**, 1418–1430 (2011).

29. J. Labbadia, R. I. Morimoto, Proteostasis and longevity: When does aging really begin? *F1000Prime Rep.* **6**, 7 (2014).

30. A. Meléndez, Z. Tallóczy, M. Seaman, E. L. Eskelinen, D. H. Hall, B. Levine, Autophagy genes are essential for dauer development and life-span extension in *C. elegans*. *Science* **301**, 1387–1391 (2003).

31. M. Manil-Segalen, E. Culetto, R. Legouis, C. Lefebvre, Interactions between endosomal maturation and autophagy: Analysis of ESCRT machinery during *Caenorhabditis elegans* development. *Methods Enzymol.* **534**, 93–118 (2014).

32. I. Saez, D. Vilchez, The mechanistic links between proteasome activity, aging and age-related diseases, *Curr. Genomics* **15**, 38–51 (2014).

33. S. A. Keith, S. K. Maddux, Y. Zhong, M. N. Chinchankar, A. A. Ferguson, A. Ghazi, A. L. Fisher, Graded proteasome dysfunction in *Caenorhabditis elegans* activates an adaptive response involving the conserved SKN-1 and ELT-2 transcription factors and the autophagy-lysosome pathway. *PLOS Genet.* **12**, e1005823 (2016).

34. Y. H. Yi, T. H. Ma, L. W. Lee, P. T. Chiou, P. H. Chen, C. M. Lee, Y. D. Chu, H. Yu, K. C. Hsiung, Y. T. Tsai, C. C. Lee, Y. S. Chang, S. P. Chan, B. C. Tan, S. J. Lo, A genetic cascade of *let-7-ncl-1-fib-1* modulates nucleolar size and rRNA pool in *Caenorhabditis elegans*. *PLOS Genet.* **11**, e1005580 (2015).

35. Y. W. Lam, A. I. Lamond, M. Mann, J. S. Andersen, Analysis of nucleolar protein dynamics reveals the nuclear degradation of ribosomal proteins. *Curr. Biol.* **17**, 749–760 (2007).

36. E. Nicolas, P. Parisot, C. Pinto-Monteiro, R. de Walque, C. De Vleeschouwer, D. L. J. Lafontaine, Involvement of human ribosomal proteins in nucleolar structure and p53-dependent nucleolar stress. *Nat. Commun.* **7**, 11390 (2016).

37. A. Sundqvist, G. Liu, A. Mirsaliotis, D. P. Xirodimas, Regulation of nucleolar signalling to p53 through NEDDylation of L11. *EMBO Rep.* **10**, 1132–1139 (2009).

38. Y. Liu, C. Deisenroth, Y. Zhang, RP-MDM2-p53 pathway: Linking ribosomal biogenesis and tumor surveillance. *Trends Cancer* **2**, 191–204 (2016).

39. M. L. Toth, T. Sigmond, E. Borsos, J. Barna, P. Erdelyi, K. Takacs-Vellai, L. Orosz, A. L. Kovacs, G. Csikos, M. Sass, T. Vellai, Longevity pathways converge on autophagy genes to regulate life span in *Caenorhabditis elegans*. *Autophagy* **4**, 330–338 (2008).

40. G. McColl, B. R. Roberts, T. L. Pukala, V. B. Kenche, C. M. Roberts, C. D. Link, T. M. Ryan, C. L. Masters, K. J. Barnham, A. I. Bush, R. A. Cherny, Utility of an improved model of amyloid-beta ($A\beta_{1-42}$) toxicity in *Caenorhabditis elegans* for drug screening for Alzheimer's disease. *Mol. Neurodegener.* **7**, 57 (2012).

41. I. Seim, S. Ma, V. N. Gladyshev, Gene expression signatures of human cell and tissue longevity. *NPJ Aging Mech. Dis.* **2**, 16014 (2016).

42. A. W. Gao, R. L. Smith, M. van Weeghel, R. Kamble, G. E. Janssens, R. H. Houtkooper, Identification of key pathways and metabolic fingerprints of longevity in *C. elegans*. *Exp. Gerontol.* **113**, 128–140 (2018).

43. F. J. Iborra, D. A. Jackson, P. R. Cook, The case for nuclear translation. *J. Cell Sci.* **117**, 5713–5720 (2004).

44. D. W. Reid, C. V. Nicchitta, The enduring enigma of nuclear translation. *J. Cell Biol.* **197**, 7–9 (2012).

45. D. M. Waldera-Lupa, F. Kalfalah, A. M. Florea, S. Sass, F. Kruse, V. Rieder, J. Tigges, E. Fritsche, J. Krutmann, H. Busch, M. Boerries, H. E. Meyer, F. Boege, F. Theis, G. Reifenberger, K. Stuhler, Proteome-wide analysis reveals an age-associated cellular phenotype of in situ aged human fibroblasts. *Aging* **6**, 856–878 (2014).

46. A. Buchwalter, M. W. Hetzer, Nucleolar expansion and elevated protein translation in premature aging. *Nat. Commun.* **8**, 328 (2017).

47. M. Derenzini, D. Trerè, A. Pession, M. Govoni, V. Sirri, P. Chieco, Nucleolar size indicates the rapidity of cell proliferation in cancer tissues. *J. Pathol.* **191**, 181–186 (2000).

48. K. Nishimura, T. Kumazawa, T. Kuroda, N. Katagiri, M. Tsuchiya, N. Goto, R. Furumai, A. Murayama, J. Yanagisawa, K. Kimura, Perturbation of ribosome biogenesis drives cells into senescence through 5S RNP-mediated p53 activation. *Cell Rep.* **10**, 1310–1323 (2015).

49. J. M. Stommel, N. D. Marchenko, G. S. Jimenez, U. M. Moll, T. J. Hope, G. M. Wahl, A leucine-rich nuclear export signal in the p53 tetramerization domain: Regulation of subcellular localization and p53 activity by NES masking. *EMBO J.* **18**, 1660–1672 (1999).

50. D. Xu, N. V. Grishin, Y. M. Chook, NESdb: A database of NES-containing CRM1 cargoes. *Mol. Biol. Cell* **23**, 3673–3676 (2012).

51. I. Topisirovic, N. Siddiqui, V. L. Lapointe, M. Trost, P. Thibault, C. Bangeranye, S. Pinol-Roma, K. L. Borden, Molecular dissection of the eukaryotic initiation factor 4E (eIF4E) export-competent RNP. *EMBO J.* **28**, 1087–1098 (2009).

52. I. Schlosser, M. Holzel, M. Murnseer, H. Burtscher, U. H. Weidle, D. Eick, A role for c-Myc in the regulation of ribosomal RNA processing. *Nucleic Acids Res.* **31**, 6148–6156 (2003).

53. J. Erales, V. Marchand, B. Panthu, S. Gillot, S. Belin, S. E. Ghayad, M. Garcia, F. Laforets, V. Marcel, A. Baudin-Baillieu, P. Bertin, Y. Coute, A. Adrait, M. Meyer, G. Therizols, M. Yusupov, O. Namy, T. Ohlmann, Y. Motorin, F. Catez, J. J. Diaz, Evidence for rRNA 2'-O-methylation plasticity: Control of intrinsic translational capabilities of human ribosomes. *Proc. Natl. Acad. Sci. U.S.A.* **114**, 12934–12939 (2017).

54. R. Huang, Y. Xu, W. Wan, X. Shou, J. Qian, Z. You, B. Liu, C. Chang, T. Zhou, J. Lippincott-Schwartz, W. Liu, Deacetylation of nuclear LC3 drives autophagy initiation under starvation. *Mol. Cell* **57**, 456–466 (2015).

55. L. J. Kraft, P. Manral, J. Dowler, A. K. Kenworthy, Nuclear LC3 associates with slowly diffusing complexes that survey the nucleolus. *Traffic* **17**, 369–399 (2016).

56. K. R. Kampen, S. O. Sulima, S. Vereecke, K. De Keersmaecker, Hallmarks of ribosomopathies. *Nucleic Acids Res.* **48**, 1013–1028 (2020).

57. I. Boria, E. Garelli, H. T. Gazda, A. Aspesi, P. Quarello, E. Pavesi, D. Ferrante, J. J. Meerpohl, M. Kartal, L. Da Costa, A. Proust, T. Leblanc, M. Simansour, N. Dahl, A. S. Frojmark, D. Pospisilova, R. Cmejla, A. H. Beggs, M. R. Sheen, M. Landowski, C. M. Buros, C. M. Clinton, L. J. Dobson, A. Vlachos, E. Atsidaftos, J. M. Lipton, S. R. Ellis, U. Ramenghi, I. Dianzani, The ribosomal basis of Diamond-Blackfan anemia: Mutation and database update. *Hum. Mutat.* **31**, 1269–1279 (2010).

58. W. Y. Huang, L. Yue, W. S. Qiu, L. W. Wang, X. H. Zhou, Y. J. Sun, Prognostic value of CRM1 in pancreas cancer. *Clin. Invest. Med.* **32**, E315 (2009).

59. F. Zhou, W. Qiu, R. Yao, J. Xiang, X. Sun, S. Liu, J. Lv, L. Yue, CRM1 is a novel independent prognostic factor for the poor prognosis of gastric carcinomas. *Med. Oncol.* **30**, 726 (2013).

60. U. H. Gandhi, W. Senapedis, E. Baloglu, T. J. Unger, A. Chari, D. Vogl, R. F. Cornell, Clinical implications of targeting XPO1-mediated nuclear export in multiple myeloma. *Clin. Lymphoma Myeloma Leuk.* **18**, 335–345 (2018).

61. A. Wahba, B. H. Rath, J. W. O'Neill, K. Camphausen, P. J. Tofilon, The XPO1 inhibitor selinexor inhibits translation and enhances the radiosensitivity of glioblastoma cells grown in vitro and in vivo. *Mol. Cancer Ther.* **17**, 1717–1726 (2018).

62. A. V. Kumar, T. G. Thakurta, M. J. Silvestrini, J. R. Johnson, R. A. Reenan, L. R. Lapierre, Give me a SINE: How selective inhibitors of nuclear export modulate autophagy and aging. *Mol. Cell. Oncol.* **5**, e1502511 (2018).

63. S. Brenner, The genetics of *Caenorhabditis elegans*. *Genetics* **77**, 71–94 (1974).

64. R. S. Kamath, J. Ahringer, Genome-wide RNAi screening in *Caenorhabditis elegans*. *Methods* **30**, 313–321 (2003).

65. J. Maciejowski, J. H. Ahn, P. G. Cipriani, D. J. Killian, A. L. Chaudhary, J. I. Lee, R. Voutev, R. C. Johnsen, D. L. Baillie, K. C. Gunsalus, D. H. Fitch, E. J. Hubbard, Autosomal genes of autosomal/X-linked duplicated gene pairs and germ-line proliferation in *Caenorhabditis elegans*. *Genetics* **169**, 1997–2011 (2005).

66. S. X. Ge, E. W. Son, R. Yao, iDEP: An integrated web application for differential expression and pathway analysis of RNA-Seq data. *BMC Bioinformatics* **19**, 534 (2018).

67. T. Kang, B. B. Boland, P. Jensen, C. Alarcon, A. Nawrocki, J. S. Grimsby, C. J. Rhodes, M. R. Larsen, Characterization of signaling pathways associated with pancreatic β-cell adaptive flexibility in compensation of obesity-linked diabetes in db/db mice. *Mol. Cell. Proteomics* **19**, 971–993 (2020).

68. T. Kang, P. Jensen, V. Solovyeva, J. R. Brewer, M. R. Larsen, Dynamic changes in the protein localization in the nuclear environment in pancreatic β-cell after brief glucose stimulation. *J. Proteome Res.* **17**, 1664–1676 (2018).

69. M. Molenaars, G. E. Janssens, E. G. Williams, A. Jongejan, J. Lan, S. Rabot, F. Joly, P. D. Moerland, B. V. Schomakers, M. Lezzerini, Y. J. Liu, M. A. McCormick, B. K. Kennedy, M. van Weeghel, A. H. C. van Kampen, R. Aebersold, A. W. MacInnes, R. H. Houtkooper, A conserved mito-cytosolic translational balance links two longevity pathways. *Cell Metab.* **31**, 549–563.e547 (2020).

70. J. Wu, X. Jiang, Y. Li, T. Zhu, J. Zhang, Z. Zhang, L. Zhang, Y. Zhang, Y. Wang, X. Zou, B. Liang, PHA-4/FoxA senses nucleolar stress to regulate lipid accumulation in *Caenorhabditis elegans*. *Nat. Commun.* **9**, 1195 (2018).
